# Taking Construction Grammar One Step Further: Families, Clusters, and Networks of Evaluative Constructions in Russian

**DOI:** 10.3389/fpsyg.2020.574353

**Published:** 2020-11-20

**Authors:** Anna Endresen, Laura A. Janda

**Affiliations:** UiT The Arctic University of Norway, Tromsø, Norway

**Keywords:** constructions, constructicon, Russian, semantics, syntax, classification

## Abstract

We present a case study of grammatical constructions and how their function in a single language (Russian) can be captured through semantic and syntactic classification. Since 2016 an on-going joint project of UiT The Arctic University of Norway and the National Research University Higher School of Economics in Moscow has been collecting and analyzing multiword grammatical constructions of Russian. The main product is the Russian Constructicon (https://site.uit.no/russian-constructicon/), which, with over two thousand two hundred constructions (and more being continuously added), is arguably the largest openly available constructicon resource for any language. The combination of this large size with depth of analysis, containing both syntactic and semantic tags, makes it possible to view the interrelation of constructions as families and to discover trends in their behavior. Our annotation includes 53 semantic tags of varying frequency, with three tags that are by far more frequent than all the rest, accounting for 30% of the entire inventory of the Russian Constructicon. These three semantic types are Assessment, Attitude, and Intensity, all of which convey a speaker’s evaluation of a topic, in contrast to most of the other tags (such as Time, Manner, and Comparison). Assessment and Attitude constructions are investigated in greater detail in this article. Secondary semantic tags reveal that negative evaluation among these two semantic types is more than twice as frequent as positive evaluation. Examples of negative evaluations are: for Assessment *VP tak sebe*, as in *Na pianino ja igraju tak sebe* “I play the piano so-so [lit. thus self]”; for Attitude *s PronPers-Gen xvatit/xvatilo (NP-Gen)*, as in *S menja xvatit* “I’m fed up [lit. from me enough].” In terms of syntax, the most frequent syntactic types of constructions in the Russian Constructicon are clausal constructions [constituting an independent clause like *s PronPers-Gen xvatit/xvatilo (NP-Gen)*] and constructions with the anchor in the role of adverbial modifier (like *VP tak sebe*). Our semantic and syntactic classification of this large body of Russian constructions makes it possible to postulate patterns of grammatical constructions constituting a radial category with central and peripheral types. Classification of large numbers of constructions reveals systematic relations that structure the grammar of a language.

## Introduction

We focus our analysis on two large and partially overlapping networks of grammatical constructions in Russian, namely the Evaluative constructions used to express Assessment and Attitude. While Assessment and Attitude will be defined and elaborated in more detail below, suffice it to say here that Assessment is an evaluation of an item external to the speaker, whereas Attitude is an expression of how the speaker feels about something. Our analysis shows how grammatical constructions function as a structured system, in which the forms of constructions are motivated by their meanings, and meanings together with syntax and anchor words connect constructions to each other.

Our aim is to represent the Assessment and Attitude networks of constructions in terms of their internal structure, as given by the families and clusters defined below. This analysis will show both hierarchical relationships within the networks of constructions, as well as lateral relationships across families, clusters, and networks. These relationships will be modeled as radial categories. While strictly speaking our conclusions are limited to this dataset, given the large size of our sample—the largest analyzed in this way thus far—we suggest that it is likely that both the remainder of Russian constructions as well as constructions in other languages can be modeled in a similar way.

Before turning to our analysis, we explain our theoretical approach in terms of construction grammar and the larger project that has given rise to this analysis, known as the Russian Constructicon, described in the section “The Russian Constructicon.” Our approach and the project provide a rich context for the analysis of the Assessment and Attitude constructions that follow in sections “A Network of Assessment Constructions: 4 Clusters and 25 Families” and “A Network of Attitude Constructions: 4 Clusters and 18 Families.” The section “Overlap of Assessment and Attitude Networks of Constructions” focuses on the ways in which the networks of Assessment and Attitude constructions overlap, and our conclusions are gathered in the section “Conclusions.” The result is a detailed demonstration of how grammatical constructions interact and in aggregate shape a linguistic system, with profound implications for the psychology of language.

### Construction Grammar and Cognitive Linguistics

Our approach is informed by construction grammar, which is itself a subfield within cognitive linguistics. Three assumptions about the nature of language characterize cognitive linguistics (Langacker, 2008; Janda, 2015). The first is the minimal assumption that language phenomena emerge from general cognitive strategies. In other words, we can explain the behavior of language in terms of what is otherwise established in the fields of neurobiology and psychology about the behavior of the brain. This assumption obviates any need for a strict division between grammar and lexicon, since both are explained by the same cognitive system. The second assumption is that generalizations about language emerge from observations of language data. Consequently, cognitive linguistics is “usage based” (Diessel, 2015; Janda, 2019), meaning that cognitive linguistics makes no strict division between “langue” and “parole,” and takes the latter as the basis for analysis. Therefore, corpora and other samples of language production are the focus of investigation. Finally, the third assumption asserts the central role of meaning for all language phenomena. Meaning is understood as grounded in human experience and elaborated by metaphor, metonymy, and blending, which supply the links in polysemous networks.

All three assumptions have direct consequences for construction grammar. In accordance with the minimal assumption, constructions cohere as a structured system following the same characteristics observed in cognitive categories, where there can be central and peripheral members (called “radial categories,” see Rosch, 1973a,b), and members of different categories can overlap and be multiply motivated because the system is strongly interconnected. Grammar and lexicon are analyzed in a unified manner. The investigation of constructions is carried out by collecting usage data, particularly from corpora, and extracting patterns that emerge from that data, and therefore construction grammar is also usage-based. Because meaning is central, the semantic pole is an essential part of the definition of a construction, explained in detail immediately below.

### Defining the Construction

Following Goldberg (1995, 2005), Croft (2001), Fried and Östman (2004), and Langacker (2008), we define the construction thus:

Constructions are entrenched language-specific form-meaning pairings available at all levels of linguistic complexity.

More specifically, a construction consists of a semantic pole (its meaning), a phonological pole (its form), and a symbolic relationship between the two poles (Langacker, 2008). An example is the Russian construction *najti-Pst NP-Acc!*^1^, literally “found X!” as in *Našli razvlečenie!* “What a bad way to amuse yourself! [lit. Found amusement!].” The semantic pole of this construction can be described thus: “The construction expresses the speaker’s dissatisfaction with the interlocutor(s), who behave incorrectly (from the speaker’s perspective) given the present situation.” The phonological pole is a past tense form of the verb *najti* “find” followed by an accusative form of a noun which serves as a direct object. This example illustrates the often non-compositional and language-specific nature of constructions. The elements of this construction (“found” + a direct object) do not in themselves indicate dissatisfaction; the whole is something that cannot be predicted on the basis of the parts^2^. This construction is specific to Russian: we do not expect to find an exact parallel in other languages, and in fact if we want to translate this construction into English, we need to render it in a variety of ways in different contexts. Three examples from the Russian National Corpus illustrate this.

(1) *– Vy, značit, emu den’gi poslali? –* ***Našli duru!*** *Ni kopejki.*
‘*–* So, in other words, you sent him money? *–* **Do you take me for a fool?! [lit. Found fool!]** Not a kopeck.’
(2) *Provodil ja Sonju, vernulsja domoj, i mama govorit: –* ***Našel krasotku!*** *Odna štukaturka.*
‘I walked Sonja to her place and when I got home, mom says: *–* **Some beauty you found yourself!! [lit. Found beauty!]** She’s just plastered [with makeup].’
(3) *Xvatit smejat’sja v biblioteke.* ***Našli mesto!***
‘Enough laughing in the library. **This is not the right place!! [lit. Found place!]**’

Note, however, that neither compositionality nor language-specificity are criteria for identifying a construction. All entrenched form-meaning pairings are constructions. The point of this example is rather to show that constructions can be non-compositional and language-specific.

From the perspective of construction grammar, the construction is the basic unit of language, and, conversely, a language is a system of constructions, also known as a “constructicon” (Fillmore, 2008; Fillmore et al., 2012). The construction is basic in the sense that it is the structure that is found throughout language, at all levels where meaning is expressed. This includes, at the minimal level, the morpheme, such as the prefix *na* (in *našli* “found”), which expresses perfective aspect^3^. Combinations of morphemes to form words are likewise constructions, as in *našli* “found,” which contains three more morphemes: *š* here indexes the root “find,” *l* marks past tense, and *i* marks plural. Our example *najti-Pst NP-Acc!* is of course a multi-word construction. Words and multi-word constructions combine to form phrases and sentences, which are also complex constructions. Further complexity is found at the discourse level with the structure of units such as requests, complaints, instructions, and the like. In its current form our Russian constructicon resource (described in more detail in the section “The Russian Constructicon” below) focuses on multi-word constructions, although in principle it would be possible to represent constructions at all levels from phonology to discourse.

The constructicon of a language is not merely an inventory. Constructions are related to each other, not just in terms of smaller parts (morphemes) being combined into units, but also in terms of relations between constructions. The idea that constructions form networks of related members was suggested by Goldberg (2005), using the example of English Subject Auxiliary Inversion, which is present in a wide range of constructions, among them questions (*Did he go?*), wishes/curses (*May you live a good life!*), negative conjuncts (*Never had she seen anything like it*), and positive rejoinders (*So do I*). Goldberg demonstrates that these constructions constitute a family based on semantic similarities, by sharing some or all of the following characteristics: the meaning of these constructions differs from that of a positive declarative sentence in that the framing is negative and/or non-declarative and/or narrowly focused and/or dependent on other clauses.

Our *najti-Pst NP-Acc!* construction belongs to a family of over a dozen constructions that signal disapproval of behavior, and in turn this family of constructions is multiply motivated, belonging to both the Assessment and the Attitude networks of constructions and thus forming a link between the two. The way in which families of constructions structure and link these two networks is described in more detail in sections “A Network of Assessment Constructions: 4 Clusters and 25 Families,” “A Network of Attitude Constructions: 4 Clusters and 18 Families,” and “Overlap of Assessment and Attitude Networks of Constructions” below. In aggregate, structured relationships like these constitute the constructicon that represents the language as a whole.

Further properties of the form and meaning of constructions that we observe in construction grammar include their idiomaticity, relationships to specific lexemes, and coercion of meaning.

Construction grammar views idiomaticity as a scalar phenomenon, with all constructions lying somewhere along a continuum between maximal idiomaticity, where a construction has fixed words and idiosyncratic syntax, to maximal schematicity, where a construction has open slots with few restrictions and typical syntactic patterns. For example, the English phrase *all of a sudden* is maximally idiomatic since it has fixed words that cannot be replaced or changed, and a syntactic pattern (quantifier + preposition + article + adjective) otherwise uncharacteristic of English. Moving slightly away from maximal idiomaticity is a phrase like *curiosity killed the cat*, where there are still absolute restrictions on the words and their forms, but the construction follows a canonical syntactic pattern, namely that of a transitive clause. Slightly further along the idiomatic <-> schematic scale we find items like *kick the bucket*, where most lemmas are fixed, but allow variation in grammatical categories, so one can use different forms of the verb, like past (*He kicked the bucket last week*) and imperative (*Go kick the bucket!*). Notice that the subject of *kick the bucket* is an open slot allowing all human (and possibly some animal) referents, and that this construction also follows the canonical transitive pattern. Also on this scale is a construction like *the X-er the Y-er* (as in *The bigger the better*), partly schematic because it has open slots albeit with some restrictions (they have to be adjectives referencing scalar qualities), but idiosyncratic syntax. Maximally schematic would be something like *NP* + *V* + *NP*, which represents a canonical transitive clause in English, consisting of only a pattern and open slots with few restrictions.

We can locate our *najti-Pst NP-Acc!* construction on the scale between idiomaticity and schematicity by observing its slots and syntax. Our construction has two slots: one slot that has a fixed lemma *najti* “find” that is restricted to past tense forms but allows variation in gender and number^4^, and one slot that is open and can be filled with any referent that can appear as a direct object of the verb. In terms of syntax, this construction is mostly aligned with standard Russian syntax for a transitive clause (with a finite verb form and a direct object in the Accusative case), but deviates slightly in that the subject is necessarily elided^5^ (in Russian it is sometimes possible to elide subjects, but not usually required to do so). In short, the *najti-Pst NP-Acc!* construction is partially idiomatic (one filled slot, restrictions on grammatical categories, requires elision of subject who is also the addressee) and partially schematic (one open slot, mostly follows usual structure of a transitive clause). Although everything on the spectrum from idiomatic to schematic is part of the constructicon of a language, our Russian Constructicon resource focuses on the items that are not at the extreme poles. In other words, we do not focus on constructions that are maximally idiomatic or maximally schematic. The reason for this is that the two poles of the continuum are already well represented in standard resources. Maximally idiomatic constructions are collected in phraseological dictionaries, and maximally schematic patterns are described in grammars. It is the constructions in between (termed “partially schematic” in Ehrlemark et al., 2016) that are the focus of our study.

Aside from the maximally schematic patterns, any given construction will usually have a special relationship to one or more lexemes. These special relationships come in two types: anchor words and common fillers. An anchor word is a fixed lemma in a construction, such as all the words in *all of a sudden* and *curiosity killed the cat*. Some anchor words participate in a large number of constructions, such as *time* in English (*time BE up*, *It’s high time VP*, *This is not the time for VPing*). Common fillers are words that typically appear in the construction, such as *bigger*, *sooner* for the first slot and *better*, *harder* for the second slot of *the X-er the Y-er* construction. Fillers are thus variables that appear in open slots in constructions. Fillers often constitute semantic groups of words, as we see in the *VP into the phone* construction, where common fillers are speaking verbs like *yell*, *mutter*, *whine*. In our *najti-Pst NP-Acc!* construction *najti* “find” is an anchor word, and some common fillers for the open slot are illustrated in examples (1)–(3).

Coercion is a phenomenon related to the non-compositional and complex meaning of constructions. Many constructions influence the meanings of the words in the construction, causing them to express meanings that they don’t otherwise have^6^. Sometimes coercion has a grammatical focus. The caused-motion construction of English can coerce an intransitive verb to express a transitive meaning, as in *The audience booed the comedian off the stage* (the caused motion construction, cf. Goldberg, 1995, p. 54), and the *NP all over* (+ *DP*) construction can coerce a count noun to be interpreted as a mass noun, as in *There was cat all over the driveway* (cf. Langacker, 2008, p. 144). More often coercion focuses on the lexical meanings and their pragmatic interpretations, as in *A(n) NP waiting to happen*, where a strong association with negatively evaluated situations causes even a neutral word like *event* to take on an ominous meaning: *an event waiting to happen* suggests danger that needs to be averted (cf. Stefanowitsch and Gries, 2003). Our *najti-Pst NP-Acc!* construction likewise coerces the meaning of its filler nouns, sarcastically forcing them to mean something like “the wrong NP, an NP I disapprove of” rather than just “NP.”

To summarize, constructions are the basic unit of language, composed of a form and a meaning and exist at all levels of language. Constructions vary along a scale from idiomatic to schematic. Constructions can invoke meanings that are not derivable from their components and can even coerce their components to express meanings that they are not usually associated with. An entire language can be modeled as a structured system of constructions, linked by meaning, syntax, and anchor words. This article is primarily focused on the last point, namely the way in which constructions constitute a language. We observe two kinds of structure in the system of the constructicon, namely hierarchical and overlapping patterns. These patterns are explored in more detail in sections “The Russian Constructicon” through “Overlap of Assessment and Attitude Networks of Constructions.”

## The Russian Constructicon

The Russian Constructicon is a free open-access electronic resource that offers a searchable database of Russian constructions accompanied with descriptions of their properties and illustrated with examples from the Russian National Corpus (www.ruscorpora.ru). The Russian Constructicon is designed for both linguists and second language learners of Russian, focusing on solid analyses of constructions as well as their annotation in terms of semantic types, syntactic patterns, morphological categories, semantic roles, and levels of language proficiency (Janda et al., 2018). Search functions make it possible to filter constructions for all of these features, as well as to access all of these features for each individual construction. The project page is available at https://site.uit.no/russian-constructicon/ (for more information on the analysis of constructions in the Russian Constructicon see Endresen et al., 2020; Janda et al., forthcoming).

Constructicons are being built for a limited number of languages: English, Swedish, German, Spanish, Brazilian Portuguese, and Japanese. The Russian Constructicon joined this movement and is currently a part of the international enterprise termed multilingual constructicography (Lyngfelt et al., 2018).

The Russian Constructicon is a joint project administered over 5 years (2016–2020) as a collaboration between two educational and research institutions: UiT The Arctic University of Norway (CLEAR research group) in Tromsø and the National Research University Higher School of Economics in Moscow (School of Linguistics). The building of this resource has been supported by two grants received from the Norwegian Agency for International Cooperation and Quality Enhancement in Higher Education [Diku, https://diku.no/en: “Constructing a Russian Constructicon” (NCM-RU-2016/10025) in 2016 and “Targeting Wordforms in Russian Language Learning” (CPRU-2017/10027) in 2017-2020].

The team working on this project includes Laura A. Janda, Tore Nesset, Anna Endresen (UiT); Ekaterina Rakhilina, Olga Lyashevskaya, Valentina Zhukova (HSE); Daria Mordashova (Institute of Linguistics, the Russian Academy of Sciences); and Francis M. Tyers (Indiana U). The website is currently under construction by Radovan Bast (Section for Digital Platform and Operation, UiT).

### Semantic Annotation of Constructions

Consistent with the assertion of cognitive linguistics that meaning plays a central role in language, we observe that the primary way in which constructions are organized is according to their semantics. With respect to the over 2,200 constructions in our Russian Constructicon resource, we find 53 meanings that yield both hierarchical and lateral (overlapping) groupings. These meanings are represented as semantic tags in the Russian Constructicon.

Semantic tags were assigned by a panel of three native speakers of Russian (including a co-author of this article) who are also linguists actively engaged in development of the content of the Russian Constructicon resource. The three taggers worked together as a panel and discussed each of over 2,200 constructions in weekly digital meetings over a period of several months. As a result, assignment of semantic and syntactic tags for individual constructions has not been a matter of individual decisions but rather an outcome of a panel decision that was often reconsidered and refined with time. As our classification of semantic and syntactic types of constructions evolved, we came back to already analyzed cases and re-analyzed them, taking into account newly gained knowledge and newly added constructions. Although any semantic interpretation of linguistic data might be regarded as subjective to some degree, we believe that using a panel of taggers helped our project to minimize the subjectivity in the analysis and secure the reliability of the outcome. This approach made it possible to control for identical and consistent understanding of the terminology used in tag-assignment and adopted by all three taggers. The terminology evolved together with the classification of constructions and the size of the database. Our system of semantic tags is to a large degree based on the categories and terminology used in typological literature [cf. the “universal grammatical set of meanings” (Plungian, 2011, p. 65) among others].

The taggers took into account corpus data as well as independent previous scholarship on individual constructions and groups of constructions. For example, in distinguishing between apprehensive and preventive constructions we followed Dobrušina (2006), recognized the types and subtypes of concession constructions according to Apresjan (1999), and consulted Rakhilina (2013) while analyzing continuative prohibitive constructions.

**FIGURE 1:**
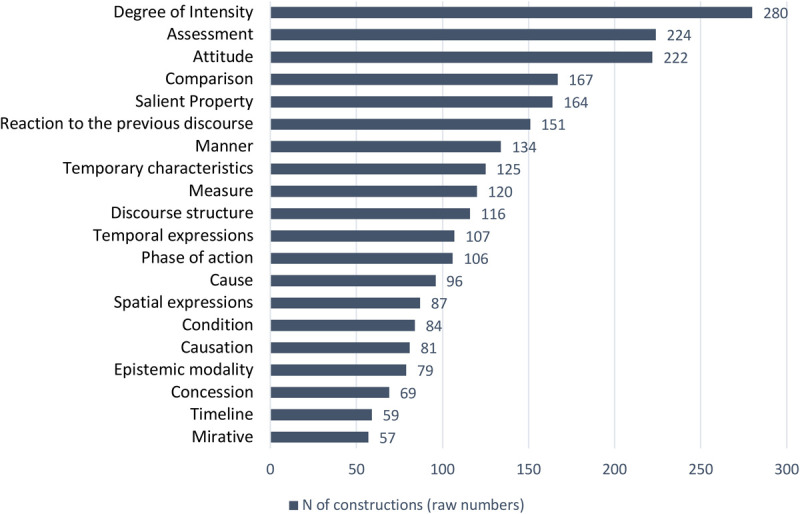
Distribution of constructions across twenty major semantic tags of top frequency.

Figure 1 displays the twenty most frequent semantic tags and their overall distribution in our database. Each of these tags is assigned to more than fifty individual constructions. The tags are listed on the left, and the bars visualize the raw numbers of constructions they describe. The numbers of constructions are provided for each bar.

The tags represented in Figure 1 refer to major semantic types of constructions. Most of these major types have an additional level of granularity represented by their subtypes that yield an overall inventory of 173 specific sub-tags. For instance, the general type Comparison has subtypes such as Inequality, Equality, Similarity, Contrast, and Imitation, following the standard typology of comparative constructions (Treis, 2018). Many constructions (over 40%) belong to more than one major semantic type, and therefore carry two or more major tags and corresponding sub-tags. Using our annotation, we can identify those semantic types of constructions that overlap with each other.

We do not exclude the possibility that when more constructions are added to the Russian Constructicon, new tags will have to be used to account for their semantics. However, the amount of data collected so far suggests that most major semantic types are already represented and identified.

Figure 1 shows that the evaluative meanings of Intensity, Assessment, and Attitude constitute the three semantic types most frequently attested in the Russian Constructicon database. They are assigned to 280, 224, and 222 constructions, respectively. Interestingly, the networks of Assessment and Attitude constructions are of approximately the same size. These networks overlap in 58 constructions that express both Assessment and Attitude.

Taking this overlap into account, we can calculate that Assessment and Attitude constructions yield 388 items, or 18% of the entire database (2,210 constructions) and thus represent a group larger than Intensity (280 constructions, 13%). As we show in sections “A Network of Assessment Constructions: 4 Clusters and 25 Families” and “A Network of Attitude Constructions: 4 Clusters and 18 Families,” both Assessment and Attitude constructions can be analyzed in terms of semantic subtypes and in terms of positive vs. negative values.

Semantic tags make it possible to subdivide the collected inventory of constructions into meaningful classes and smaller groups of constructions, turning an initial list into a structured network. Those constructions that belong to the same semantic subtype often share some syntactic (syntactic function in a clause, the structure of the anchor part) and structural properties (such as negation, inversion, or reduplication). Such groups of constructions form families, and families form clusters, as we detail in the next subsection.

### Hierarchical Patterns Within the Constructicon

We find hierarchical patterns within the Russian Constructicon, where we can identify three levels, which we term “Families,” “Clusters,” and “Networks.”

Families are smaller groups, usually of 2–9 constructions. Table 1 displays three families of constructions used to express evaluation of objects and actions in the cluster *Assessment in relation to norms/expectations* of the Assessment network.

**TABLE 1:** Three families of Assessment constructions.

**Name of construction**	**Short Illustration**	**English [ + literal translation]**
**Family 1: Evaluation of an object as important**		
NP-Nom Cop v cene^7^	*Ran’še družba byla v cene*	“Friendship used to be appreciated [lit. earlier friendship was in price].”
NP-Nom Cop v počete	*Fiziki u nas v počete*	“Physicists are highly respected here [lit. physicists by us in honor].”
NP-Nom imet’ (Adj) značenie	*A kakoe èto imeet značenije, ždali ètu junuju ledi ili ne ždali?*	“Does it matter [lit. what this has meaning] whether they waited for the young lady or not?”
NP-Nom ne imet’ (Adj) značenija	*Den’gi uže ne imejut značenija*	“Money plays no role anymore [lit. already not have meaning]”
NP-Nom igrat’ Adj rol’	*Odežda igraet važnuju rol’ na sobesedovanii*	“Clothes play an important role at a job interview”
NP-Nom ne igrat’ (nikakoj) roli	*Èto obstojatel’stvo ne sygralo v ego sud’be nikakoj roli*	“This circumstance made no difference in his life [lit. did not play in his fate no role].”
VP NP-Acc s rukami (i nogami)	*V sekciju po plavaniju menja brali s rukami i nogami – ja pokazyvala neploxie rezul’taty.*	“I was easily accepted into the swimming sports club [lit. they took me with arms and legs], because I was good at it.”
NP s bol’šoj bukvy	*On vrač s bol’šoj bukvy*	“He is a very good doctor [lit. spelled with a capital letter]”
NP-Nom Cop u PronPoss-Gen nog	*Ves’ mir u našix nog*	“We have power/control over others [lit. the whole world is at our feet]”
**Family 2: Evaluation of an activity as worth doing**		
NP-Nom togo stoit’	*Poezdka v Afriku togo stoit*	“The trip to Africa is worth taking [lit. trip that costs]”
NP-Nom stoit’ desjati NP-Gen	*Odin čas obščenija s uvlečennym i znajuščim čelovekom stoit desjati pročitannyx knig*	“An hour of talking to an enthusiastic and competent person equals the effect of having read 10 books [lit. costs ten read books]”
**Family 3: Evaluation of an object as unimportant**		
vsego liš’ NP	*Ona vsego liš’ medsestra*	“She is just a nurse [lit. all only nurse]”
vsego-navsego NP	*Èto byl vsego-navsego staryj divan*	“This was merely [lit. all on all] an old sofa”
Cl, (a) tak, Cl	*Ona mne ne nravilas’, a tak, balovstvo odno*	“I didn’t like her, you see [lit. and thus], I was just having fun”
(s)dat’sja-Pst PronPers-Dat ètot NP-Nom!	*Dalsja tebe ètot neudačnik!*	“There’s a loser for you! [lit. gave-self to you that loser]”
sovsem ešče NP	*On sovsem ešče mal’čik*	“He is just [lit. entirely yet] a boy”
Cl, čto s NP-Gen Cop vzjat’?	*On daže ne zakončil školu, čto s nego vzjat’?*	“He did not even graduate, what can you expect of him? [lit. what from him take]”
čto/čego s NP-Ins Cop govorit’/sporit’, Cl	*čto s nim govorit’, on vse ravno sdelaet po-svoemu*	“There’s no point talking with him [lit. what with him talk], he will just do what he wants anyway”
NP-Nom predstavljat’ iz sebja NP-Acc	*Ty iz sebja voobšče ničego ne predstavljaeš’!*	“You’re completely irrelevant! [lit. you from yourself in general nothing not represent]”

In Table 1, notice that the constructions in each family are nearly synonymous, and some of them also share similar syntactic structure and anchor words. The constructions in Family 1 all evaluate an object as important, though this evaluation can be negated as well. In contrast, the constructions in Family 3 necessarily evaluate the object as inadequate. Family 2 is specialized to the evaluation of activities. Syntactically we see some parallels, for example in Family 1 there are two constructions consisting of an NP followed by the preposition *v* and a noun in the Locative case (*NP-Nom Cop v cene* and *NP-Nom Cop v počete*). Also in Family 1 we see five constructions exhibiting the canonical syntax of a transitive clause [*NP-Nom ne igrat’ (nikakoj) roli*, *NP-Nom imet’ (Adj) značenie*, *NP-Nom ne imet’ (Adj) značenija*, *NP-Nom igrat’ Adj rol’*, *VP NP-Acc s rukami (i nogami)*]. Both constructions in Family 2 use the Genitive case to signal quantification. Family 3 is syntactically somewhat diverse, but contains three constructions with adverbial phrases modifying NPs (*vsego liš’ NP*, *vsego-navsego NP*, *sovsem ešče NP*). In terms of anchor words, the collocations *imet’ značenie* “have meaning” and *igrat’ rol’* “play role” are important in Family 1; in Family 2 both constructions contain the verb *stoit’ “*cost,” and in Family 3 we see that forms of the determiner *ves’* “all” recur.

### Expansion of the Russian Constructicon

Organization of constructions in terms of families, clusters and networks helped us to expand the scope of the Russian Constructicon by filling out the families of constructions.

**FIGURE 2:**
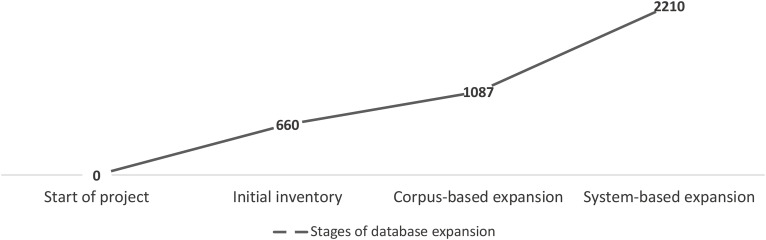
Stages of database expansion and the cumulative size of the database at each stage.

Figure 2 visualizes the key stages of database expansion: start of the project, initial inventory, corpus-based expansion, and system-based expansion, showing how many constructions the database contained at each stage.

An initial inventory of 660 constructions was amassed manually from a variety of sources including textbooks for learners of Russian and scholarly literature on Russian constructions, as well as a crowd-sourced Google spreadsheet. We then added 407 constructions using manual text analysis, by culling from running texts of various kinds, particularly those that contain dialogs and spoken discourse, as well as an automatically extracted list of highly frequent collocations attested in the Russian National Corpus. Thus overall, 1,087 constructions were added through corpus-based means. This method does not target semantic or syntactic types, but relies instead on the unpredictable appearance of constructions in running text. Subsequently we worked in a different direction and applied a method of system-based expansion of the database. This method entailed examining semantic families of constructions already in the database and searching for synonyms, antonyms, and related constructions containing the same or similar anchor words in order to fill gaps in each family (mostly using native intuition). We therefore classified the first 1,087 collected constructions into meaningful families and clusters and added the missing constructions to each family. This process yielded 1,123 new items, and the database reached the current size of 2,210 constructions. Comparing the 407 corpus-based added items vs. 1,123 system-based added items shows that the latter methodology turned out to be almost three times more effective (2.8 times, to be precise). In other words, our efficiency in discovering additional constructions was aided by the classification: once we knew what to look for, constructions became easier to find.

Our work on semantic groups of constructions turned what initially was a list of unrelated items into a structured inventory of constructions, where we have plenty of relevant information on both hierarchical and lateral relations among and across constructions. We can now show how families form clusters and how these groupings overlap with each other by sharing some of the same members. Moreover, we are now in a position to estimate the amount of overlap for various semantic types and syntactic patterns of constructions and to show how semantic types and syntactic patterns of constructions can relate to each other.

## A Network of Assessment Constructions: 4 Clusters and 25 Families

### Overview

Assessment constructions express evaluation of an item external to the speaker. This item can be understood as an object of Assessment, using the word “object” in a broad sense. An object can be a physical object, or an animate participant in a situation, or a situation itself. For example, Assessment constructions can evaluate someone’s appearance or intellectual capacity. We analyze Assessment constructions in terms of semantic types and in terms of the polarity values they carry, that is positive vs. negative Assessment.

Overall, out of 224 (100%) constructions, there are almost twice as many constructions that encode negative Assessment as opposed to those that express positive Assessment (109 vs. 57 items, or 49% vs. 25%). A set of 58 constructions (26%) can express either of the two values depending on the lexical fillers of their slots (as in *na redkost’ Adj/Adv* used in both *na redkost’ umnyj “*unusually smart” and *na redkost’ lenivyj* “unusually lazy [lit. on rareness]”) and the possibility of negation (as in *VP (ne) k mestu* “do something (not) to the point [lit. (not) to place],” e.g., *Ty očen’ k mestu èto skazala* “You said it very much to the point” vs. *On ljubut ne k mestu pošutit’ “*He tends to tell inappropriate jokes”).

Arutjunova (1988) provides a detailed overview of several influential theories of Assessment, showing how they matter for understanding linguistic data, summarizing works by Aristotle, Kant, Perry, Hare, Wittgenstein, Vendler, and many others. Value is a complex category that has been discussed broadly in philosophy, ethics, and logic (cf. theory of value, discussion of moral value, the nature of goodness and other issues). Following “The Varieties of Goodness” by von Wright (1963) and applying his taxonomy to data on Russian value predicates (mostly adjectival), Arutjunova (1988, p. 75) suggests that axiological meanings expressed linguistically can be broken down into two major types: General Assessment (“obščaja ocenka”) and Specific Assessment (“častnaja ocenka”). General Assessment is an overall, undifferentiated Assessment that evaluates an object holistically, approaching it as a whole. General Assessment is expressed by the adjectives that denote “good” or “bad” and their synonyms that vary in terms of expressivity and stylistics (e.g., *prekrasnyj* “wonderful,” *zamečatel’nyj* “excellent,” *durnoj* “nasty,” etc.). By contrast, Specific Assessment evaluates an object not as a whole but from one of various possible perspectives, focusing on a single property of an object. For example, Specific Assessment can refer to evaluation of physical qualities (like shape or smell) or the usefulness of an object. Having re-classified and somewhat simplified the taxonomy of values described by von Wright (1963), Arutjunova suggests that Specific Assessment can be further subdivided into Sensory, Ethical and Aesthetical, and Rationalistic types.

In our analysis of Assessment constructions attested in Russian, we adopt the distinction of General vs. Specific Assessment discussed in von Wright (1963) and Arutjunova (1988), but we group the specific subtypes of the latter in a different way, as motivated by the data we analyzed^8^. In this section we identify several crucial semantic types of Assessment constructions in Russian and model their relationship as a radial category of families and clusters that form a network of constructions.

### A Radial Category Model

**FIGURE 3:**
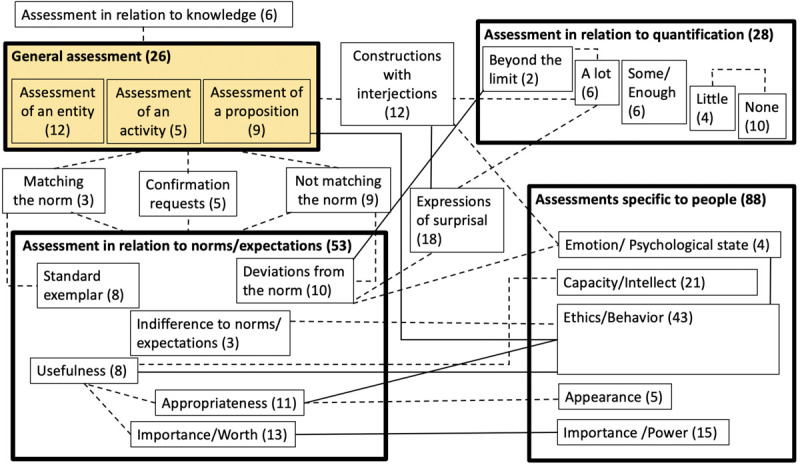
A radial category model of the network of Assessment constructions.

Figure 3 presents a radial category model of Assessment constructions, showing how they form families and clusters, and how these units are related to each other within this network. Large boxes visualize clusters of constructions, smaller boxes represent families, and lines between boxes connect clusters and families that are closely related in terms of semantics or/and involve the same individual constructions. Solid lines indicate both conceptual closeness and overlaps between the groups (observed when constructions are associated with more than one family or cluster). Dashed lines link the groups that exhibit conceptual closeness only. The thickness of the box contour and the size of the box represent the type frequency which is likely indicative of relative entrenchment of the cluster in the network. The visualization is determined by these observed relationships. Numbers in parentheses are type frequencies for each family and cluster, that is the number of individual constructions from our dataset that belong to each unit. The classification of constructions across these families and clusters results from our analysis of data and has been verified against the intuitions of two additional taggers.

Figure 3 shows that Assessment has several dimensions. We distinguish between General Assessment, Assessment in relation to quantification, Assessments specific to people, and Assessment in relation to norms/expectations. The two latter clusters are the most prominent in terms of type frequency. Assessment related to knowledge is a distinct type of Assessment that is encoded by a family of six constructions. Because it does not belong to any of the four large clusters, we represent it as a separate structural unit of the network. Many families belong to more than one cluster at the same time: *Matching the norm, Confirmation Requests, Not matching the norm, Constructions with interjections* and *Expressions of surprisal*. We call them “transitional” and represent them by boxes placed outside the clusters. These families are connected by lines to those clusters where they belong.

General assessment is conceptually the most basic and prototypical type of assessment and is most intensively connected with all other clusters, a further indication of its prototypicality (Lewandowska-Tomaszczyk, 2007). In Figure 3, the prototypical cluster is shaded.

Figure 3 represents transitional families that belong to two or more clusters. Table 2 provides type frequencies for each cluster, both without and including transitional families.

**TABLE 2:** Distributions of Assessment constructions across the four clusters.

**Cluster**	**Number of constructions**	**Number of constructions including transitional families**
General assessment	26	26 + 3 + 5 + 9 + 12 = 55
Assessment in relation to norms/expectations	53	53 + 3 + 5 + 9 + 18 = 88
Assessment specific to people	88	88 + 12 = 100
Assessment in relation to quantification	28	28 + 12 + 18 = 58

The total is larger than 224 constructions because some of these constructions belong to multiple families.

Table 2 makes it possible to estimate the degree of overlap between the four clusters, that is the number of constructions that belong to more than one unit of this network is 71 constructions, yielding 32% of our sample of Assessment constructions (where 224 = 100%)^9^.

In what follows we present each cluster and briefly characterize the families it contains.

### General Assessment

General assessment is the most basic type of assessment not restricted to a certain domain and expressed by 26 constructions in our database. General Assessment refers to an overall evaluation of an object (in the broad sense) as a whole. Each construction in this cluster contains evaluative lexemes that denote “good” or “bad.” For example, in the construction *dela (u NP-Gen) Cop ploxi* (as in *Dela u nego ploxi* “Things go wrong for him [lit. affairs by him bad]”), the anchor includes the adjective *ploxoj* “bad” that clearly encodes negative evaluation of a situation.

Russian offers a range of various partially schematic expressions that often carry colloquial flavor and are more or less semantically equivalent to the “neutral” standard lexemes *xorošij* “good” and *ploxoj* “bad.” Syntactically, such constructions represent a variety of patterns, mostly populating three syntactic subtypes: (1) constructions with a predicative anchor part, (2) constructions where the anchor functions as an adverbial modifier, and (3) biclausal constructions with matrix predicates in the main clause. Each of these syntactic types is compatible with both positive and negative evaluative semantics, as illustrated in the following three paragraphs. These subtypes form families of constructions that we term *Assessment of an entity, Assessment of an activity*, and *Assessment of a proposition* respectively.

#### The Family *Assessment of an Entity*

Predicative phrases with positive assessment include constructions like *NP-Nom Cop ničego (takoj-Nom)* (as in *professor on byl ničego* “He was an okay professor [lit. nothing]”). Examples of predicative phrases with negative evaluation come from the constructions *NP-Nom Cop ne očen’* (as in *Dlja stojanki mesto ne očen’* “The place is not so good for parking [lit. not very]”), *NP-Nom Cop tak sebe* (as in *kartina tak sebe* “the painting is so-so [lit. that self]”), and *NP-Nom Cop ne axti (kakoj-Nom/kakoj Adj-Nom/kakoj Noun-Nom)* (as in *Iz-za vetra skorost’ byla ne axti “*Because of the wind the speed was not so good [lit. not ah]”).

#### The Family *Assessment of an Activity*

Constructions with the anchor in the role of adverbial modifier include similar expressions encoding positive assessment: *VP na slavu* (as in *Prazdnik udalsja na slavu “*The party was a success [lit. on glory]”), and *VP ničego* (as in *Kormili v našej stolovoj ničego “*The food in our canteen was okay [lit. They fed in our canteen nothing]”). Negative assessment is expressed in adverbial constructions like *VP tak sebe* (as in *Na pianino ja igraju tak sebe “*I play the piano not so well [lit. that self]”) and *VP-Ipfv počem zrja* (as in *Paša rugaetsja počem zrja každyj den’* “Paša (diminutive from Pavel) argues indiscriminately [lit. how-much in vain] every day.”

#### The Family *Assessment of a Proposition*

Biclausal constructions of General Assessment contain matrix predicates that are elaborated in a subordinate clause. For example, in the construction *PronPoss sčast’je, čto Cl* (as in *Ego sčast’je, čto rejs zaderžali, inače by ne popal na samolet “He was lucky* [lit. his happiness] that the flight was delayed, otherwise he would not have gotten on the plane”), the matrix is the anchor noun *sčast’je ‘*happiness’, and it requires a dependent clause that explains the grounds for the evaluation. Another good example of this pattern comes from the construction *NP-Nom Cop, konečno, NP-Nom, čto Cl* (as in *Ja, konečno, durak, čto poslušalsja tebja* “I am, of course, a fool, that I followed your advice”), where the matrix predicate is not the anchor but a slot that can be filled with evaluative nouns of either positive or negative value: *molodec* and *umnica*, both meaning “attaboy,” or *durak* and *glupec*, both referring to a “fool.”

Previous scholarship suggested that General Assessment predicates tend to be semantically deficient and therefore require context to support the evaluative judgment (Arutjunova, 1988, p. 92–94). Our data support this claim in that the biclausal constructions with evaluative matrix predicates attach a subordinate clause that substantiates and specifies the meaning of the main clause. Another way to compensate for the informative deficiency of evaluative predicates is to describe the domain of goodness/badness of an object via the instrumental case. As an example, consider the construction *NP-Nom Cop xorošij-Short/ploxoj-Short NP-Ins* (as in *èti mesta xoroši svoimi lesami “*These places are good in terms of their forests [lit. by their forests],” where the noun *lesa “*forests” is used in the instrumental case) (cf. Arutjunova, 1988, p. 94 for discussion).

Summing up, General Assessment contains subgroups of constructions that are defined in terms of both semantic and syntactic properties. On the one hand, semantics is expressed in the syntactic structure, and on the other hand, the syntax predetermines nuances of semantics. Thus, we arrive at a more or less homogeneous group of constructions at the intersection of semantics and syntax, taking both of these characteristics into account.

### Assessment in Relation to Norms/Expectations

Previous studies of value predicates showed that the concepts of the norm, the standard, and the expectations associated with them play a crucial role in motivating the linguistic expressions of Assessment. In this sense, Assessment constructions serve as a type of reference point constructions, and the latter are considered pervasive in human cognition (cf. Rosch, 1977, 1978; Langacker, 2008, p. 83–85). The concept of the norm refers to cultural and social conventions that constitute an idealized model of the world that people often rely on (cf. Arutjunova, 1988, p. 202). In cognitive linguistics, this idea has been discussed in terms of Idealized Cognitive Models (Lakoff, 1987) that structure our background knowledge, and in terms of “mental spaces” (Fauconnier, 1985) that represent cognitive constructs of potential worlds relevant for human communication. When evaluating, speakers tend to compare the evaluated object to their idealized cognitive model, which functions as a standard. The idea of what is normal suggests to the speaker what to expect. A failure to match the expectations can cause a surprise, often an unpleasant one. Usually, matching the norm yields positive assessment, whereas deviations from the norm motivate negative assessment.

We find that these concepts are crucial for understanding a prominent group of constructions that encode Assessment in terms of what is normal, standard, and/or expected. Here we can observe the association of positive vs. negative values and matching vs. non-matching of the norm in three families of constructions. These families are transitional in nature and can be best understood as belonging to two clusters: General Assessment and Assessment in relation to norms.

The first family is termed *Matching the norm* and includes three constructions with anchor words that refer to norms and standards: *VP kak nado* (as in *Otec gotovil jaičnicu kak nado “*Father fried the eggs just right [lit. like need]”), *VP kak sleduet* (as in *On rabotal kak sleduet “*He worked properly [lit. like follows]”), and *NP-Nom Cop čto nado* (as in *Prazdnik čto nado “*The party is super-duper [lit. what need]”). All three constructions express positive evaluation motivated by the semantics of fitting into the standard, expected and proper performance.

The other family is termed *Not matching the norm* and includes nine constructions that encode negative evaluation. Constructions of this type formally resemble general holistic positive evaluation, but in fact mean the opposite, ironically pointing to deviations from the standard/norm. Examples include *xorošij-Short NP-Nom!* (as in *Xoroš učenyj! “*The opposite of a good scholar! [lit. Good scholar!]”), *tot ešče NP* (as in *To ešče udovol’stvie! “*A notorious [lit. that yet] pleasure!”), *tože mne NP-Nom!* (as in *Tože mne geroj! “*A false/pseudo- [lit. too to me] hero!”). Most constructions of this semantic type share a certain syntactic pattern: they represent exclamatory clausal statements that assign a name to an object of evaluation that does not deserve this name. The exclamatory intonation emphasizes the speaker’s resentment about the mismatch between the evaluated object and the name or status it has been assigned: e.g., [*ešče (i)] NP-Nom nazyvaetsja*, as in *Moloka ne daet. Korova nazyvaetsja! “*It gives no milk. What a bad cow it is! [lit. cow is-called].”

A third transitional family of constructions contains *Confirmation requests* that seek to establish whether an object corresponds to the normal representative of a category X. Syntactically, such constructions share the patterns of rhetorical questions like *razve ne NP-Nom Cop?* (as in *Razve ne krasota? “*Isn’t it a beauty? [lit. really not beauty]”) and *Cl, čem Cop ne NP-Nom* (as in *Prismotris’ k Miše. Čem ne ženix?* “Take a better look at Miša. As good a bridegroom as any/In what respect is he not a bridegroom? [lit. which not bridegroom]”). Although formally the speaker is questioning whether the object matches the norm, the form of these questions indicates that the assumption behind them is that the object clearly does so, and positive evaluation is conveyed by establishing this correspondence between the object and the norm.

Apart from these transitional families, the cluster Assessment in relation to norms/expectations also includes the families *Deviations from the norm* and *Standard exemplar*. Closely related to the concept of the norm and expectedness are the families *Appropriateness, Importance/Worth, Usefulness*, and *Indifference to norms/expectations*.

The family *Deviations from the norm* includes 10 constructions that specify in what respect the norm is not matched. For example, many constructions in this group refer to a large size or a large number of objects, and this relates them to the Quantification cluster: consider the construction *NP-Gen.Pl Cop vyše kryši/golovy* (*Problem vyše kryši “*Problems through the roof [lit. higher roof]”). Some constructions in this family refer to deviations from the norm that come with positive evaluation, like *ničego sebe (takoj) NP* (as in *Ničego sebe mašina!* “Wow, what a car! [lit. nothing itself car!]”). Other constructions specify deviations that are compatible with both positive and negative views of the situation. For instance, the construction *na redkost’ Adj/Adv* “unusually [lit. on rareness!]” supports both types of uses: *na redkost’ krasiv “*unusually pretty” and *na redkost’ glup “*unusually stupid.”

The family *Standard exemplar* is a group of eight constructions that evaluate an object as the most prominent of its kind, the best example of a category. Most constructions in this family share a non-trivial structural property: a reduplicative nominal pattern, where the noun is repeated in the same or a different grammatical case. Examples of such constructions are *NP-Nom Cop vsem Noun-Dat.Pl* ∼*Noun-Nom* (as in *Vsem borščam boršč “*The best vegetable soup of all [lit. to all soups soup]”) and *NP-Nom Cop Noun-Nom* ∼*Noun-Ins* (as in *On takoj glupyj, durak durakom “*He is so stupid, a fool times two [lit. fool by-fool],” cf. a detailed discussion of this construction in Janda et al. (2020) and references therein). A closely related subset of constructions compares the object to the standard and indicates that the object is so standard that this makes it average, unremarkable, ordinary, and unimpressive. The construction *(èto Cop) Noun-Nom kak* ∼*Noun-Nom* (as in *Xleb kak xleb “*Just normal bread [lit. bread like bread]”) evaluates the standard exemplar positively, whereas the construction *(nu) XP i* ∼*XP* (as in *Byl u teti Maši kot. Nu kot i kot. Ničego osobennogo “*Aunt Maria had a cat. Just an ordinary cat, nothing special [lit. well cat and cat]”) suggests that the speaker evaluates the standard-like nature of the cat to be uninteresting and even boring.

The family of constructions termed *Appropriateness* conveys a rationalistic evaluation of whether an object fits the situation. Most of these constructions contain predicative phrases that can alternatively modify verb phrases and can also be negated: compare *NP (ne) v temu* (as in *Tvoi zamečanija sejčas sovsem ne v temu “*Your remarks are now completely out of place [lit. not in topic]”) and *VP (ne) v temu* (as in *On skazal èto očen’ v temu! “*He said it very much on point [lit. in topic]”). Similarly used prepositional phrases include *(ne) k mestu* [lit. (not) to place], *(ne) po delu* [lit. (not) on business], and *(ne) v kassu* [lit. (not) in cash register] all referring to well-fitting vs. ill-fitting in the conversation, as well as *v točku* [lit. into point] meaning “to the point” and *mimo kassy* [lit. past cash register] meaning “beside the point.”

The three families of constructions listed above in Table 1 refer to the concepts of *Importance/Worth* and *Importance/Power* and evaluate an object as important vs. unimportant and an activity as worth doing. By assessing an object as important, the speaker assigns it a certain value (e.g., *NP-Nom Cop v cene*, as in *Ran’še družba byla v cene “*Friendship used to be appreciated [lit. was in price]”), that can or cannot play a role *(NP-Nom igrat’ Adj rol’ “*play a role”), matter, and affect the situation (*NP-Nom imet’ (Adj) značenie “*matter [lit. have meaning]”). Importance motivates positive evaluation, and lack of value implies negative evaluation of an object. In those constructions that assign value to animate referents, the concept of Importance transforms into Power and Respect: consider the constructions *NP-Nom Cop u PronPoss-Gen nog* (as in *Ves’ mir u našix nog “*We have power over others [lit. the whole world is at our feet]”) and *NP-Nom Cop v počete* (as in *Fiziki u nas v počete “*physicists are highly respected here [lit. physicists by us in honor]”) that connect the *Importance/Worth* family to the cluster Assessment specific to people (family *Importance/Power*). Note that most constructions in the three *Importance* families (Table 1) are specific either to inanimate referents (including abstract notions like factors, properties, relationships) or to animate referents: compare *NP-Nom Cop v cene “*appreciated” (for inanimates) vs. *NP-Nom Cop v počete “*respected” (for animates) accordingly. By contrast, a few constructions allow both types of fillers, like the pattern *NP s bol’šoj bukvy “*very good [lit. with capital letter]” that can be encountered in positive evaluations of persons of certain professions (e.g., *vrač/učitel’/aktrisa s bol’šoj bukvy “*a highly professional and talented doctor/teacher/actress”) or evaluations of certain occasions (e.g., *delo/moment/igra s bol’šoj bukvy “*highly important and critical business/moment/game”). Similarly, in the family of Assessment constructions that evaluate an object as unimportant, the first three constructions (*vsego liš’ NP; vsego-navsego NP; Cl, (a) tak, Cl*, all meaning “merely”) can refer to both animate and inanimate referents, whereas the remaining four constructions (e.g., *sovsem ešče NP* “merely”; *Cl, čto s NP-Gen Cop vzjat’? “*what can you expect of?”) encode evaluation of a person and thus rather belong to the cluster Assessment specific to people. In this light, representation of all interrelations between the constructions in a network like Assessment can hardly be adequately depicted in a two-dimensional model like Figure 3, which should be treated as an approximation of the real picture^10^. Rather, one should keep in mind that analysis allows for different levels of granularity that account for the fact that certain subsets of constructions within a single family can belong to several clusters at the same time (in this case, the clusters Assessment in relation to norms/expectations and Assessment specific to people). This only proves the point of a radial category model that recognizes the legitimacy of multiple overlaps and the lack of rigid categorical distinctions between the established groups of data.

Another important overlap can be observed between the families encoding *Importance* on the one hand and the *Usefulness* family on the other hand. Both constructions that evaluate activities (e.g., *NP-Nom togo stoit’*, as in *Poezdka v Afriku togo stoit “*The trip to Africa is worth taking [lit. trip that costs]”) and constructions that evaluate objects and persons (*VP NP-Acc s rukami (i nogami)* [lit. with arms and legs]) suggest that the value of an object or activity is often established on the basis of the speaker’s personal benefit from using this object or performing this activity. One can benefit from something one can effectively use.

The *Usefulness* family of constructions determines the so-called teleological evaluation of an object and defines whether an object can be of any use. The construction *vidavšij vidy NP* (as in *Na vidavšem vidy velosipede ja poexal dal’še “*I went biking on the weather-beaten bicycle [lit. having seen sights bicycle]”) can carry either positive or negative assessment depending on the context: it can either refer to an old and well-worn object in case of negative evaluation or, by contrast, to an object that the speaker has confidence in, values and relishes. Another curious construction in this family is *(NP-Dat) NP-Nom (ne) katit’* (as in *Mne takoj argument ne katit “*For me this point does not work [lit. not rolls]”). This construction has a strong colloquial flavor and shows that usefulness can be assessed on the basis of appropriateness, thus conceptually relating the two categories and the two families. Objects that are appraised as appropriate are “supported” by standard expectations, they tend to be useful and positively evaluated. By contrast, constructions like *zrja/naprasno VP* (as in *Zrja staraeš’sja “*You strive in vain”) carry negative assessment, suggesting that there is no need in doing X, as this is not useful for the situation.

A separate family of constructions denote *Indifference to norms/expectations*. However, in terms of assessment, such constructions are not neutral but clearly negative, as in the following example: *VP PronInt popalo* (e.g., *Vasja šlet pis’ma komu popalo “*Vasja sends letters to every Tom, Dick or Harry [lit. to-someone it-fell]”). In this example, the first comer, or any random person is evaluated negatively and the whole activity of dealing with people indiscriminately also receives a negative evaluation.

We have seen that the cluster Assessment in relation to norms/expectations is connected not only to General Assessment, but also to Assessment specific to people (*Importance/Worth* and *Importance/Power* families) and to Assessment in relation to quantification (*Deviations from the norm* family). We will now examine each of these clusters in turn.

### Assessment Specific to People

Assessment specific to people is a large cluster that contains several families of constructions. The most important groups here involve *Capacity/Intellect* and *Ethics/Behavior*, with smaller groups for *Importance/Power*, *Appearance*, and *Emotion/Psychological state.*

The family *Capacity/Intellect* contains twenty-one constructions that assess someone’s ability to perform a certain activity or deal with a certain subject or academic discipline. Most of these constructions refer to intellectual abilities and encode positive evaluation of the capacity itself, and any kind of activity can fill the slot.

Syntactically, we can observe a rich variety of patterns including anchor predicative phrases in *NP-Nom Cop gorazd VP-Inf/na NP-Acc* (as in *On na vydumki gorazd “*He is very inventive [lit. strong on inventions]”) and *NP-Nom Cop NP-Nom VP-Inf* (as in *On master gotovit’ “*He is good at cooking [lit. expert cook]”); anchor light verbs in *NP-Nom znat’ tolk v NP-Loc* (as in *On znaet tolk v nastol’nyx igrax “*He is an expert in board-games [lit. He knows sense in board-games]”); anchor adverbials in *VP na pjaterku/pjat’ ballov/otlično* (as in *znat’ matematiku na pjaterku* “know math at the highest level [lit. on five]”); and clauses like *NP-Nom VP-Inf Cop ne durak* (as in *On vypit’ ne durak “*He can drink well [lit. have-a-drink not fool]”).

Semantically, prominent strategies of referring to good intellectual abilities employ conceptual blending (Fauconnier and Turner, 2002) of producing ideas and cooking food that we see in the metaphorical construction *u NP-Gen golova varit’* (as in *U Peti golova varit – s nim možno imet’ delo “*Peter has his head screwed on right [lit. by Peter head stews], so one can do business with him.” Other constructions denote measuring intellectual abilities in terms of having enough sense to perform an activity: e.g., *(NP-Dat/u NP-Gen) xvatit’ NP-Gen VP-Inf*, as in *U nee xvatilo uma priostanovit’ supruga “*She had the wisdom to stop her husband [lit. had enough cleverness]”). An alternative strategy is stating whether one needs to borrow some wisdom (*NP-Gen NP-Dat Cop ne zanimat’*, as in *Xitrosti emu ne zanimat’ “*He does not need to borrow any cunning”) or whether wisdom is an inalienable possession (*NP-Gen u NP-Gen ne otnimeš’/Cop ne otnjat’*, as in *Talanta u nego ne otnjat’ “*One cannot take his talent from him”).

Negative evaluation of intellectual abilities is expressed by constructions like *u NP-Gen NP-Nom xromat’* (as in *U brata sil’no xromaet geografija “*The brother does not have a good handle of geography/has problems with geography [lit. by brother strongly limps geography]”).

Conceptually, the family *Capacity/Intellect* is related to *Usefulness* since persons with strong intellectual capacity can also be useful.

The largest family in the cluster Assessment specific to people is termed *Ethics/Behavior* and contains constructions that evaluate someone’s behavior in terms of general ethical or personal standards. This group of constructions is closely related to *Appropriateness* and mostly contains constructions that carry negative evaluation. Syntactically, constructions in this family are comprised of either mono-clausal or biclausal statements, often flavored with an exclamatory intonation of indignant criticism. The above-mentioned construction *najti-Pst NP-Acc!* “found X!” (as in *Našli razvlečenie!* “What a bad way to amuse yourself! [lit. found amusement!]” belongs here, along with numerous other clausal constructions like *delat’ PronPers-Dat Cop nečego!* (as in *Delat’ tebe nečego! “*You should not be doing this/Don’t you have anything better to do than this!” [lit. do to-you nothing!]”), the construction *nado že Cop (NP-Dat) VP-Inf* (as in *Nado že bylo svjazat’sja s takimi ljud’mi!* “And it had to happen so that you got involved with such (bad) people! [lit. needed well was connect with such people!]”), etc. Biclausal constructions denote not only negative evaluation of someone’s activity or behavior, but they also name a positively evaluated alternative behavior that one could have been doing instead: compare the construction *net čtoby/by VP-Inf, Cl* (as in *Net čtoby podoždat’, on ušel bez nas!* “Instead of having waited for us, he just left! [lit. no in-order wait]”) and the construction *čem by VP, VP (by)* (as in *čem by učit’sja, on guljaet!* “Instead of being busy with his studies, he is outdoors! [lit. than could study, he takes a walk]”). Some constructions in this family convey positive or negative evaluation through evaluative anchor words, and thus relate this family to the General Assessment cluster: e.g., *(NP-Dat) ne grex Cop i VP-Pfv.Inf*, as in *Teper’ ne grex nam i otdoxnut’ “*Now there is no harm in taking a rest [lit. not sin us and rest].”

Regarding the *Importance/Power* family, see discussion in section “Assessment in Relation to Norms/Expectations.”

A family of five constructions expresses aesthetic assessment of someone’s *Appearance*. Some constructions evaluate whether a piece of clothing fits the outfit and overall look of a person, and thus conceptually connects the *Appearance* family to the *Appropriateness* family discussed above. We encounter both predicative phrases as anchors of constructions *NP-Nom Cop NP-Dat k licu* (as in *Sinee plat’je bylo ej k licu “*The dark blue dress was becoming to her [lit. to face]”) and *NP-Nom Cop (NP-Dat/dlja NP-Gen) v samyj raz* (as in *Dlja kukly èta šapka v samyj raz “*The hat is the right fit for the doll [lit. in same one time]”), and certain anchor verbs of motion like *podxodit’* “approach by walking” and *idti* “walk”: e.g., *NP-Dat idti XP* (as in *Ej idet èta pričeska* “This hairdo looks good on her [lit. to her goes hairdo]”).

*Emotion/Psychological state* is a family of constructions that assess psychological properties or an emotional state of a person. Such constructions tend to indicate those properties that stand outside of the norm. This concerns both temporary characteristics like emotional states (e.g., *NP-Nom Cop sam ne svoj* (as in *Papa segodnja sam ne svoj “*Dad is not himself today [lit. oneself not one’s own]”) and constant characteristics like personality type or temper (e.g., *NP-Nom Cop sebe na ume*, as in *Vasja sebe na ume, nikogda ne govorit vsej pravdy “*Vasya has his own agenda [lit. to oneself on mind], he never tells the whole/full truth”).

### Assessment in Relation to Quantification

The cluster of Assessment in relation to Quantification constructions serves to relate the Assessment network to other constructions that encode quantification and degree of intensity. This cluster includes several families distinguished on the basis of different degrees, or quantities, of a certain property. The relevant degrees form a scale and include: *None, Little, Some/Enough, A lot*, and *Beyond the limit*. A prominent group of constructions includes various *Expressions of Surprisal*. Overall, constructions in this cluster show that qualitative evaluation (positive vs. negative) is motivated by quantitative assessment.

In the context of the conceptual metaphor MORE IS BETTER (Lakoff and Johnson, 1980), the zero level of a property (“none”) is associated with negative evaluation: compare constructions like *NP na nule* (as in *Immunitet na nule “*Immunity is absent/does not function/is at the zero level” [lit. on zero], *NP-Ins (tut/tam) i ne paxnut’* (as in *Naukoj tut i ne paxnet “*Science is nowhere near here” [lit. with science here and not smells], and *nikakoj PronPers Cop ne XP* (as in *Nikakoj on ne genij “*He is not a genius at all” [lit. none he not genius].

A small degree of a property (“little”) is encoded in patterns like *ne takoj už i Adj* (as in *ne takoj už i strašnyj “*not so frightening”).

A larger amount of a property (“some”) is often positively evaluated, if it is enough for performing an activity: *NP-Nom Cop dostatočno Adj, čtoby VP-Inf*, as in *On dostatočno vzroslyj, čtoby ponjat’ èto “*He is old enough to understand this.”

Denoting a high degree of a property (“a lot”) often comes along with positive evaluation: *čertovski Adj/Adv* (as in *On čertovski umen “*He is drop-dead smart [lit. devilishly smart],” *vo vsex otnošenijax XP* (as in *Novyj spektakl’ byl vo vsex otnošenijax udačnym “*The new performance was successful in all respects”). However, intensifiers are compatible with both positive and negative evaluations. A highly prominent strategy of encoding high degree of a property in evaluative constructions is to use an interrogative pronoun in exclamative function^11^, as in *kakov Cop NP-Nom!* (as in *Kakov podlec! “*What a rascal! [lit. which rascal]”). Often, a pronoun is combined with additional intensifiers: *(možno) s uma sojti kakoj Adj* (as in *Sumka u nee s uma sojti kakaja dorogaja! “*Her bag is crazy expensive! [lit. bag by her from-mind-depart what expensive]”). Such exclamatory clauses with pronouns tend to imply surprisal due to a greater amount of the property than expected, and in this regard such constructions are transitional to the cluster Assessment in relation to norms/expectations. This connection is even more evident in the *Beyond the limit* family, in constructions like *VP/Adj sverx mery* (as in *On odaren sverx mery “*He is talented above measure”).

Some evaluative constructions that encode high degree of a property contain both a pronoun and an interjection that accompany the evaluative statement. Whereas the pronoun takes the role of intensifier, the interjection often clearly specifies whether the construction carries positive or negative evaluation. For example, the patterns *iš’, kakoj Adj-Nom Cop* (as in *Iš’, kakoj veselyj! “*How inappropriately glad he is!”) and *fu, kakoj NP-Nom Cop!* (as in *Fu, kakaja gadost’! “*Yuck, what a disgusting thing!”) always carry negative assessment, whereas the constructions *ux ty, kakoj/kak XP!* (as in *Ux ty, kakuju rybu pojmali! “*Wow, what a fish we have caught!”) and *aj da NP-Nom!* (as in *Aj da geroj! “*What a hero!”) obligatorily encode positive evaluation. This family of constructions can be considered transitional between the cluster Assessment in relation to quantification and the cluster of General assessment, as it equally belongs to both clusters. Also, because interjections encode specific emotions (e.g., *ux ty* expresses surprise, *aj da* encodes admiration and praise, *fu* stands for disgust, etc.), one can argue that these constructions are additionally motivated by the cluster Assessment specific to people that contains the family *Emotion/Psychological state*.

### Assessment in Relation to Knowledge

A distinct family of six constructions stands outside of the clusters discussed above and encodes *Assessment in relation to knowledge*. These constructions can evaluate an object, a situation participant, time, or space depending on whether it is known or unknown information. All constructions in this family convey negative evaluation arguably motivated by the fact that something is unknown and unspecified. Representative examples come from the constructions like *bog vest’ PronInt* (as in *Oni prinesli v pakete bog vest’ čto “*They brought who knows what in the bag” [lit. God knows what]), *neznamo PronInt* (as in *Neznamo kak ja vernulsja domoj “*I came home without knowing how” [lit. not-known how]), *ne NP kakoj-nibud’* (as in *My ne bomži kakie-nibud’! “*We are not some homeless people!”), etc.

### Summary of Assessment Constructions

Assessment motivates a highly complex network of constructions in Russian organized both hierarchically and horizontally. Hierarchically we observe over two dozen families of constructions which are internally relatively homogenous, sharing semantics and often syntactic patterns as well. Most of these families can be grouped into clusters which in turn give structure to the overall network. Horizontally we see relationships between families and between clusters motivated both by constructions with allegiances to multiple families, and via conceptual similarity. For example, three families connect these two clusters: General Assessment and Assessment in relation to norms/expectations. Conceptual similarity is observed among constructions that focus on usefulness, importance/worth, intellectual capacity, and appropriateness. Examination of a large number of constructions makes it possible to spot trends and confirm claims of previous scholars, for example about the tendency for General Assessment to be expressed in a biclausal construction, and the skewed polarity of assessment. The latter tendency toward negative polarity is even more pronounced in the network of Attitude constructions which is the topic of the section “A Network of Attitude Constructions: 4 Clusters and 18 Families.”

## A Network of Attitude Constructions: 4 Clusters and 18 Families

### Overview

Whereas Assessment constructions evaluate an item external to the speaker, Attitude constructions, by contrast, refer to evaluation of the speaker’s internal state of mind or internal emotional approach taken toward a situation. In other words, Attitude constructions express how the speaker feels about something, what standpoint he or she takes, what the speaker’s personal perspective on a subject or a situation is.

As in the case of Assessment constructions, we analyze Attitude patterns both in terms of semantic types and in terms of polarity values (positive vs. negative Attitude).

In terms of semantic types, we found that Attitude constructions are highly diverse but can still be grouped under general and specific domains. For example, we distinguish between clusters such as Emotional Attitude and Mental Attitude, and at a more granular level we recognize families of constructions encoding Skepticism, Perplexity, Confidence, etc. (see subsection “A Radial Category Model” for details).

In terms of polarity values, we found that the vast majority of Attitude constructions in our dataset carry negative evaluation. Over 72% (159 out of 222 items) of constructions in this network are used to encode negative Attitude, whereas only 18% (40 items) of constructions refer to positive Attitude. The remaining 10% of Attitude constructions are neutral for polarity, which is determined instead by other factors (see below). For example, the construction *Cl, ne vopros* (as in *Ja vse sdelaju, ne vopros “*I will do everything, this is not a problem [lit. not question]”) can only express positive attitude and willingness to perform an activity, whereas the construction *NP-Dat Cop ne do NP-Gen* (as in *Mne ne do uborki “*I am not going to tidy up now (assuming that I have a lot of other things on my plate or I have no time for it right now) [lit. to me not to tidying]”) is restricted to imply only negative attitude and lack of willingness to perform an activity.

The observed distribution (72% negative vs. 18% positive) might suggest that a large part of the network of Attitude constructions serves the need to express a range of subtle differences of speaker’s attitudes and/or express approximately the same type of attitude in a variety of different ways, ranging in terms of politeness vs. strictness, transparency vs. opacity, etc. Comparing the distribution of positive vs. negative values in Attitude and Assessment networks, we observe that the relative proportion of constructions encoding negative Attitude is higher than that of negative Assessment constructions (compare 72% Attitude vs. 49% Assessment, respectively). However, the difference in positive value rates is not that dramatic: positive Attitude in 18% vs. positive Assessment in 25% of each of the two relevant datasets, respectively. This finding suggests that Attitude constructions as a network are even more negative than Assessment constructions that specify all possible nuances of deviations from the norm, expectations, and standards. Negative attitude constructions clearly predominate in our dataset.

We observe that only 10% (22 items) of Attitude constructions (as opposed to 26% of Assessment constructions yielding 58 individual items) can carry either positive or negative evaluation depending on the fillers, possibility of negation, or a broader context. For example, the same construction *kak NP-Nom Cop Adj-Short, čto Cl!* can be used to express both positive and negative Attitude, depending on the filler of the slot: compare *Kak ja rad, čto ty vernulas’! “*I am so glad that you came back!” vs. *Kak ja zol, čto svjazalsja s ètoj firmoj! “*I am so angry that I got involved with this agency!” In a similar way, a negated version of a construction can express the opposite polarity value, as in *(NP-Dat) oxota/neoxota Cop VP-Inf*: e.g., *Mne spat’ oxota “*I want to sleep” vs. *Mne rabotat’ neoxota “*I do not want to work.” In some cases, interpretation of the attitude value expressed by a construction is only possible in a broader context or might even be not entirely appropriate, as in the case of *kak že NP-Dat Cop ne VP-Inf?* (e.g., *Kak že mne ne pomnit’? “*How could I fail to remember (given this situation) [lit. how well me not remember]?”) that refers to the lack of choice and can be seen as a type of attitude associated with neither of the two polarity values.

Attitude constructions are very diverse in terms of semantic and syntactic types and complex in terms of their relationships and multiple overlaps with each other, as we show in the next section.

### A Radial Category Model

**FIGURE 4:**
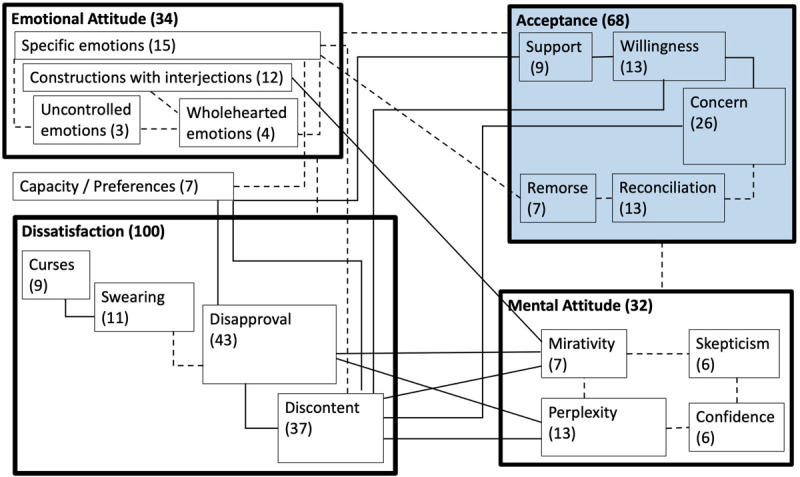
A radial category model of the network of Attitude constructions.

We model the network of Attitude constructions as a radial category visualized in Figure 4. This model accounts for the major semantic types of Attitude constructions as well as minor relevant distinctions and their relations with one another. We adopt the same manner of representation of the radial category structure as in the section “A Radial Category Model.”

Figure 4 shows that Attitude constructions form a complex network that consists of four large clusters and eighteen families. Large boxes visualize clusters of constructions termed Acceptance, Dissatisfaction, Emotional Attitude, and Mental Attitude. Smaller boxes represent families inside these clusters as well as one family that does not belong to any of these clusters, namely *Capacity/Preferences*. Solid lines connect those units that overlap (contain constructions that belong to more than one family), and dashed lines indicate conceptual connections. Shading highlights the Acceptance cluster as the most prototypical in this network. We observe that this cluster is conceptually the most general one and it provides motivation links to all remaining clusters. The Dissatisfaction cluster, although more numerous, is a specific case, a “negated” version of Acceptance. Numbers in parentheses indicate type frequencies for each unit of this network. Note that the total is larger than 222 constructions because some constructions belong to more than one family. This concerns only 12 constructions (5% of the Attitude dataset), showing that the amount of overlap between the families of this network is smaller than that of the Assessment network, estimated at 32% (cf. the section “A Radial Category Model”).

In the following subsections we present each cluster and characterize each family of the Attitude network.

### Acceptance

Constructions of the *Acceptance* cluster convey the meaning that the speaker more or less accepts the situation. This cluster includes the families *Support, Willingness, Concern, Reconciliation*, and *Remorse.* Each of these families suggests additional semantic nuances to the general meaning of *Acceptance* and has certain tendencies in selecting syntactic structures and anchor lexemes.

Constructions that form the *Support* family express whether the speaker takes someone’s side, shares someone’s opinion, or promotes a certain idea that aligns with his or her own interests or views. For example, the constructions *NP-Nom Cop (ne) protiv NP-Gen* (as in *Ja protiv škol’noj formy! “*I do not support having a school uniform [lit. against uniform]”) and *NP-Nom Cop za NP-Acc* (as in *Ja za revoljuciju “*I support the idea of revolution [lit. for revolution]” usually encode the speaker’s attitude to abstract concepts, institutions, regulations, and situations. By contrast, the construction *NP-Nom Cop na PronPoss-Loc storone* (as in *V ètom spore ja na vašej storone “*In this argument I am on your side”) encodes a positive attitude toward someone’s opinion or executed strategy. Syntactically, these constructions usually employ predicative prepositional phrases and nominal patterns.

The *Willingness* family of Attitude constructions carries the meaning that the speaker is willing or unwilling to perform an activity. Some constructions in this group encode this meaning transparently by means of the anchor word *xotet’* “want”: e.g., *NP-Nom i slyšat’ ne xotet’ o NP-Loc* (as in *On i slyšat’ ne xočet o poezdke! “*He does not want to even hear about the trip [lit. and hear not want about trip]”). Other constructions employ derivatives of the verb *xotet’* “want,” namely the nouns *oxota* “willingness” and *neoxota* “reluctance,” as well as a synonymous noun *len’* “laziness.” These nouns perform a predicative function and govern an infinitive denoting an activity in the constructions *(NP-Dat) oxota/neoxota Cop VP-Inf* (as in *Mne spat’ oxota “*I want to sleep [lit. to me willingness sleep]”) and *(NP-Dat) len’ Cop VP-Inf* (as in *Mne len’ gotovit’* “I do not want to cook [lit. to me laziness cook]”). Less semantically transparent are the structures that convey the semantics of unwillingness via predicative prepositional phrases like *v lom “*a bummer” (consider the construction *NP-Dat Cop v lom VP-Inf*, as in *Maše idti v magazin bylo v lom “*Maria did not want to go to the store [lit. to Maria walk in store was in bummer]”) and *ne do NP-Gen* “not to X” (*NP-Dat Cop ne do NP-Gen*, as in *Mne ne do uborki “*I am not going to tidy up now [lit. to me not to tidying]”). Infinitival constructions encode the (un)willing subject in the dative case, thus morphologically suggesting that an unenthusiastic attitude is rather a state that “happens” to the subject and this lack of agentivity and control arguably implies lack of responsibility that the speaker is willing to take for the attitude in question (see Divjak and Janda, 2015 for detailed discussion). An interesting case in this regard is the construction *(u NP-Gen) ruki ne doxodit’ VP-Inf* that does not openly claim the unwillingness to perform an activity and instead transfers the responsibility for the speaker’s failure to achieve a result to the lack of the right circumstances: e.g., *Ruki ne doxodjat kryšu počinit’ “*I did not get around to fixing the roof [lit. arms not arrive roof fix].”

In contrast to an entire armory of means to express a lack of enthusiasm about an activity, a smaller subgroup of constructions denotes the speaker’s readiness for active participation and positive attitude toward it. This type can be illustrated with constructions like *VP-Inf Cop (da/voobšče/da voobšče) ne vopros* (as in *Postroit’ dom – ne vopros “*Building a house – sure! [lit. to build house not question]”) and *Cl, bez problem/voprosov* (as in *Ja vse sdelaju, bez problem! “*I will do everything, no problem! [lit. without problems]”).

*Concern* is a large family of twenty-six Attitude constructions that encode the speaker’s indifference or concern about the situation. Most constructions refer to unconcern and express negative attitude: e.g., *malo (li) PronInt VP* (as in *Malo li čto on poprosit! “*Whatever he asks for, it does not matter [lit. little what he will ask].” Many constructions contain the anchor word *delo* “business” or *vnimanie “*attention”: compare *komu kakoe delo Cop do NP-Gen* (as in *Komu kakoe delo do tvoej problemy “*No one cares about your problem [lit. whom what business to your problem]”) and *Cl, a NP-Nom ne obraščat’ vnimanija* (as in *Oni tam derutsja, a ona ne obraščaet vnimanija “*They are fighting, but she does not pay attention [lit. not turn attention]”). Syntactically, this family is a diverse and non-homogeneous group that includes adverbial patterns like *VP-Imp postol’ku-poskol’ku* (as in *Ego interesuet èto postol’ku-poskol’ku* “He is mostly uninterested in this issue [lit. insomuch in-how-much]”), predicative patterns like *NP-Dat Cop vse ravno* (as in *Mne vse ravno “*It is all the same to me [lit. me everything same]”), with the majority of clausal constructions like *čto PronPers-Dat NP-Nom* (e.g., *čto mne dožd’ “*It does not matter to me whether it rains [lit. what to me rain]”), and biclausal syntactic structures like *nu i čto, čto XP* (as in *Nu i čto, čto xolodno “*What’s the big deal if it is cold [lit. well and what, that cold]”). Often, constructions of this family blend together, producing structures like *èkzameny ne èkzameny, emu vse ravno* “*Exams or not, it does not matter to him* [lit. exams not exams, to him all same],” where we encounter a combination of the construction *XP ne* ∼*XP, Cl* and the construction *NP-Dat Cop vse ravno.*

The *Reconciliation* family of constructions suggests that the speaker accepts the situation even though it is not desirable and often appears to be out of the speaker’s control. We observe this semantics in many biclausal constructions, where one clause names the situation, whereas the other clause indicates the speaker’s attitude. By means of example consider the construction *Cl (i/no) (tut) (už) ničego (s ètim) (NP-Dat/NP-Nom) ne podelat’* (as in *On uezžaet, i tut ničego ne podelaeš’* “He leaves, there is nothing to do about it [lit. and here nothing not do]”) and the construction *čto už tam, Cl* (as in *čto už tam, moja vina “*What shall I say [lit. what there], it is my fault”). By using the former construction, the speaker suggests that nothing can be done to change the situation, whereas the latter construction states that nothing can be said to argue against the truth. Most constructions in the *Reconciliation* family express positive attitude of the speaker (e.g., *čto s PronPers-Ins (budeš’) delat’!^12^* (e.g., *Opjat’ ty ves’ grjaznyj! Čto s toboj delat’! “*You are all dirty again! It can’t be helped! [lit. what with you do!]”), or/and lack of choice, as we see in the expressions like *nekuda devat’sja, Cl^13^* (as in *Nekuda devat’sja, nužno emu pomoč’ “*There is no way out [lit. nowhere get], we have to help him”). It is implied that, having no choice, the speaker adopts a strategy that is the only one acceptable in the given situation or in the speaker’s view, as illustrated with a similar construction *(NP-Dat) nel’zja Cop ne VP-Inf* (as in *Nel’zja bylo ne soglasit’sja s nim togda “*It was impossible to disagree [lit. impossible was not agree] with him in that moment.”

Additionally, the *Reconciliation* family includes a notable structural type of various reduplicative patterns, where the same lexeme is repeatedly used in the same or a different morphological form. A good example comes from the construction *XP tak* ∼*XP* (as in *Sup tak sup “*If I should eat the soup, I will do so [lit. soup then soup]”) and a synonymous pattern *XP značit* ∼*XP* (as in *Dieta – značit dieta! “*If I should go on a diet then I will do so! [lit. diet means diet]”). Even less semantically transparent is a similar reduplicative construction *(nu) XP i* ∼*XP* (as in *Včera ja poterjal kol’co. Nu poterjal i poterjal, ne nado dumat’ o ploxom “*Yesterday I lost a ring. It happened, whatever [lit. well lost and lost], no need to think about bad things”).

The *Remorse* family of constructions provides the speaker with various ways to express sadness and regret about what the speaker (or another participant) has done or about the state of affairs in general. An example of the former comes from the construction in *čert (PronPers-Acc) dernul VP-Inf* (as in *čert menja dernul pošutit’ “*I don’t know what got into me that I made that joke [lit. demon pulled me joke]”), whereas the latter can be illustrated with the construction *žal’ Cop, Cl*, as in *Žal’, nogi promokli “*It is a pity that [someone’s] feet got drenched.” Remorse constructions are used in situations when the speaker has to report on something unpleasant or undesired for him- or herself and/or their interlocutor. Therefore the role of such constructions is often to mitigate the negative effect of the upcoming information by expressing the speaker’s sympathy and compassion with the interlocutor. Syntactically, many of these constructions contain a parenthetical expression that introduces a clause [e.g., *k (PronPoss/Adj) sožaleniju, Cl*, as in *K sožaleniju, my ne možem vam pomoč’ “*Unfortunately [lit. to regret], we cannot help you”] or a matrix predicate (e.g., *beda Cop, čto Cl*, as in *Beda, čto on ne prišel “*It is a disaster that he did not come”), or an interjection (e.g., *uvy, Cl!*, as in *Uvy, koncert otmenili “*Too bad, the concert is canceled”). By expressing regret, the speaker arguably takes partial responsibility for the negative information he/she reports on, and therefore the attitude encoded in these constructions is best captured by the term Remorse.

The Acceptance cluster thus gathers constructions that represent conceptually related nuances. Support is something that is offered when someone is willing to act, and willingness is related to a show of concern. Reconciliation and remorse are two types of acceptance in the face of difficulties.

### Dissatisfaction

The largest group of Attitude constructions expresses various kinds of *Dissatisfaction*. All constructions of this cluster carry negative evaluation and constitute four distinct families that form a rising scale of negativity: *Discontent* > *Disapproval* > *Swearing* > *Curse*.

The thirty-seven constructions that form the *Discontent* family share the semantics of relatively mild dissatisfaction on the part of the speaker regarding the entire situation: e.g., *Cl, a NP-Nom VP-Imp!* (as in *On ušel domoj, a ja opjat’ peredelyvaj vse posle nego “*He went home, and I again have to redo [lit. I redo] everything after him.” By using *Discontent* constructions, the speaker fulfills the need to complain about an unsatisfactory state of affairs, often claiming that there are so many problems that having one more additional problem is even worse. Therefore, many constructions in this family contain anchor words that denote “shortage” or “enough”: compare *(NP-Dat) tol’ko NP-Gen (ešče) ne xvatalo!* (as in *Tol’ko doždja ne xvatalo! “*Rain is the last thing I needed! [lit. only rain not was enough]”).

The *Disapproval* family comprises 43 constructions that encode both the speaker’s strong negative Attitude and negative Assessment of someone’s behavior. This group of constructions is the home of the above-mentioned construction *najti-Pst NP-Acc!* “found X!” (as in *Našli razvlečenie!* “What a bad way to amuse yourself! [lit. found amusement!]”) and constitutes a large zone of overlap connecting the two networks, as described in section “Assessment Specific to People” (family *Ethics/Behavior* of Assessment constructions).

*Swearing* constructions form a family of 11 constructions that mark an even more negative Attitude of the speaker toward the situation. Swearing constructions included in the Russian Constructicon contain anchor swear words like *čert “*demon” or its derivatives: e.g., *kakogo čerta Cl!* (as in *Kakogo čerta zdes’ tak grjazno! “*Why the devil is it so dirty here?”).

*Curse* constructions form a distinct family of nine constructions that denote the highest degree of negative Attitude. Curse constructions do not necessarily contain swear words but obligatorily carry the intention of harming someone or something: *Cl, bud’ PronPers-Nom prokljatyj-Short!* (as in *Opjat’ èti komary, bud’ oni prokljaty! “*Again these mosquitos, damn them [lit. be they damned]!”).

### Mental Attitude

The Cluster termed *Mental Attitude* is formed by constructions denoting Attitude motivated by the speaker’s knowledge or expectations. This cluster comprises four families: *Skepticism, Confidence, Perplexity*, and *Mirativity.*

A *Skeptical* attitude on the part of the speaker is conveyed by constructions that are used in speaker’s responses to a statement made by the conversation partner. All of these constructions express different shades of disagreement with the previous discourse. Many of these constructions employ a peculiar syntactic pattern: they repeat the key part of the interlocutor’s statement and frame it with an Attitude construction. Consider such an “echo”-pattern in the construction *skažeš’/skažete tože – XP* (as in*—On takoj xorošij! – Skažeš’ tože – “xorošij”! “*– He is so nice! – Come on! How can you say that! [lit. you say too *–* “nice”].” The construction *vot ešče, XP!* (as in *– Da ty vljublena v nego! – Vot ešče, vljublena! “*– You seem to be in love with him! – In love? No way! [lit. here more, enamored]”) is organized in a similar way: it repeats the exact quote of the preceding problematic statement made by the interlocutor and argues against it. Another example comes from the construction *rasskazyvaj/rasskazyvajte, Cl* (as in – *U nas ne bylo deneg. – Rasskazyvaj, ne bylo deneg!* “– We had no money. – Tell me another, “had no money”! [lit. tell, not was money]”) that expresses the speaker’s doubts and distrust.

The *Confidence* family aggregates six constructions that express the speaker’s certainty about his or her knowledge. All constructions in this family contain the anchor words *znat’* “know” or *dumat’* “think”: *PronPers-Nom PronPers-Acc znat’-Prs, Cl* (as in – *Ja tebja znaju, ty vse razboltaeš’! “*I know you, you are going to blab it all”) and *Tak PronPers-Nom i dumat’/znat’-Pst, (čto) Cl* (as in – *Tak ja i dumal, čto ty menja obmaneš’ “*I knew [lit. so I and thought] that you were going to deceive me”).

The *Perplexity* family is represented by thirteen constructions that encode the speaker’s uncertainty about the cause of a situation or the actions of another participant. In terms of syntax, all these constructions are questions: e.g., *da i PronInt VP?* (as in *Da i gde ego sejčas najdeš’?* “And where can one find him now? [lit. and where find]”). Often *Perplexity* constructions can additionally signal the speaker’s discontent, and in this regard they are related to the *Discontent* family of the *Dissatisfaction* cluster: *čto že NP-Nom VP?* (as in *čto že on sidit?* “Why is he sitting (and not acting)? [lit. what well he sits]”).

The *Mirativity* family of seven Attitude constructions encodes the speaker’s surprise caused by new and unexpected information (see DeLancey, 1997; Aikhenvald, 2012 for discussion of the term). The construction *vot tebe i raz/na: Cl* can express both positive and negative attitude of the speaker (as in *Vot tebe i na: u nee tri dočki i dvoe synovej! “*There you are [lit. here to you take]! She has three daughters and two sons!”). Some mirative constructions encode surprise accompanied with frustration: compare negative evaluation in e.g., *(NP-Nom VP, čto/kazalos’ by) Cl/XP, an net!* (as in *Ja nadejalas’, čto den’gi vernut, an net! “*I hoped that I could get the money back, but nothing came out of it [lit. on the contrary no!]”). These constructions relate the *Mirativity* family to the *Discontent* family in the *Dissatisfaction* cluster. Syntactically, all constructions in this family contain a clause.

We observe that each family in the *Mental Attitude* cluster employs a characteristic syntactic pattern. Conceptually, we can establish connections between these groups: *Skepticism* is related to *Confidence*; *Confidence* is the opposite of *Perplexity*; and *Perplexity* is close to *Mirativity.*

### Emotional Attitude

A cluster of constructions denoting Emotional attitude is related to other clusters through their families of *Remorse, Discontent*, and *Mirativity* constructions. The *Emotional attitude* cluster is highly diverse, but we can distinguish three major semantic subtypes that form families: constructions that name specific emotional attitudes, constructions that refer to strong uncontrolled emotions, and constructions that emphasize the depth or scope of the feeling. This cluster also contains a family of *Constructions with interjections* discussed in the section “Assessment in Relation to Quantification.”

Constructions expressing specific emotional attitudes (*Specific emotions* family) often include anchor words that name the emotion within a nominal pattern: e.g., *VP na radost’ NP-Dat* (as in *Na radost’ detjam vypal sneg “*Much to the children’s delight [lit. on gladness/joy to children], it snowed”) and *k užasu/sčast’ju NP-Gen, Cl* (as in *k užasu mamy, vse moroženoe rastajalo “*Much to mom’s horror, all the ice cream melted”). However, there are some constructions that specialize in expressing emotional attitude even without anchor words naming an emotional state. By means of example consider the reduplicative construction *NP-Dat Noun-Nom Cop ne (v)* ∼*Noun-Acc (bez NP-Gen)* (as in *Devočkam radost’ ne v radost’ “*For the girls their joy was not real rejoicing [lit. gladness not in gladness]”)^14^, that indicates impossibility to enjoy a certain emotional state because of some external interference.

Constructions that refer to strong uncontrolled emotions (the *Uncontrolled emotions* family) can be illustrated with such structures with light verbs as *NP-Nom vyjti iz sebja* (as in *Načal’nik vyšel iz sebja “*The boss lost his temper [lit. walked out from self]”) and *NP-Nom poterjat’ golovu (ot NP-Gen)* (as in *On poterjal golovu ot sčast’ja “*He went crazy for happiness [lit. lost head from happiness]”).

Constructions that emphasize the depth or scope of a feeling in the *Wholehearted emotion* family tend to have an adverbial modifier function: compare the synonymous constructions *VP do glubiny duši* (as in *On obidelsja do glubiny duši “*He took offense to the bottom of his heart [lit. to depth of soul]”) and *VP vsem serdcem* (as in *Ja vsem serdcem perežival za nee “*I was wholeheartedly [lit. by entire heart] distressed for her”), etc.

The *Emotional attitude* cluster serves to relate the Attitude network of constructions to the Assessment network. This cluster is conceptually similar to the *Emotion/Psychological state* family of the cluster *Assessment specific to people* (recall section “Assessment Specific to People”).

### Capacity/Preferences

A family that does not belong to any of the Attitude clusters is formed by constructions that denote *Capacity/Preferences*: e.g., *NP-Nom Cop s NP-Ins na* “*vy*” (as in *Ja s texnikoj na* “*vy*” *“*I am not friends [lit. on ‘you’] with technical equipment”). Being capable to deal with something motivates the attitude of feeling comfortable or uncomfortable with a certain activity: *XP èto Cop ne PronPers-Nom* (as in *Xodit’ po teatram – èto ne moe “*Going to the theaters is not my strong point”).

### Summary of Attitude Constructions

While the Attitude network is somewhat less complex than the Assessment network, the overall types of structure are the same. Attitude constructions comprise a multiply interconnected system, with both hierarchical relationships that join families into clusters and clusters into the network, as well as horizontal relations across families and clusters linked via shared constructions and similar concepts. And while both networks are biased toward negative evaluations, the Attitude network is even more strongly skewed in the negative direction.

## Overlap of Assessment and Attitude Networks of Constructions

**FIGURE 5:**
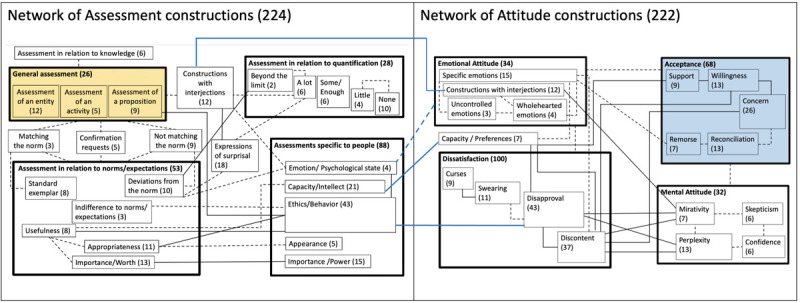
Overlap of Assessment and Attitude networks.

In addition to the horizontal relationships we have mapped out within both the Assessment and the Attitude networks, we find strong horizontal relationships across the two networks, which is not surprising given that one’s assessment of something or someone can influence one’s attitude to that something or someone. This conceptual proximity is realized also in a number of constructions that are multiply motivated by both networks. As diagrammed in Figure 5, there is overlap across the two networks in three families of constructions, namely constructions signaling assessment of an attitude toward the capacity of people, their negatively evaluated behavior, and emotional attitudes, as detailed below. The families in question are linked with solid blue lines. Conceptual closeness is indicated with the dashed blue line that connects the Emotional Attitude cluster of constructions with the *Emotion/Psychological state* family of Assessment constructions.

The largest portion of this overlap is contributed by forty-three constructions that simultaneously belong to the *Ethics/Behavior* family of Assessment and the *Disapproval* family of the Attitude network. We observe that negative evaluation of someone’s behavior mostly supports negative attitude to such behavior, as we observe in the construction *najti-Pst NP-Acc!*, literally “found X!” as in *Našli razvlečenie!* “What a bad way to amuse yourself! [lit. Found amusement!].”

Second, both networks contain a family of 12 constructions with interjections, where the NP conveys the Assessment, whereas the interjection expresses emotional attitude of the speaker: e.g., *fu, kakoj NP-Nom Cop!* (as in *Fu, kakaja gadost’! “*Yuck, what a disgusting thing!”).

Finally, three constructions simultaneously belong to *Capacity/Intellect* family of Assessment and *Capacity/Preferences* family of Attitude, including the construction *NP-Nom Cop s NP-Ins na* “*vy*” (as in *Ja s texnikoj na* “*vy*” *“*I am not friends [lit. on ‘you’] with technical equipment”). This example illustrates that depending on the filler of the NP-Nom slot the semantics of constructions can shift toward Assessment or Attitude: if the referent is the speaker, then the construction conveys his or her attitude to a certain type of activity (in this case: dealing with technical equipment), whereas, if the referent is another participant, the construction is rather used to encode Assessment of his or her abilities to deal with a certain object named by *NP-Ins*, as in this example from the Russian National Corpus:

(4) *Nepravda, čto vse* ***ženščiny s texnikoj na* “*vy.*”**
‘It is not true that all **women are unable to deal well with [lit. on “you”] technical equipment’**.

Overall, the overlap of the two networks amounts to 58 constructions (26% of each network).

## Conclusion

Our case study of Assessment and Attitude constructions in Russian is part of the first large-scale study of the structure of a constructicon of any language and represents an advance in the mapping of semantic fields expressed by grammatical constructions. Whereas the semantics of lexemes that express evaluation has been subjected to classification (cf. [Bibr B39][Bibr B41]), this is the first study of a large number of constructions that serve this function. And whereas there have been numerous detailed studies of individual constructions and smaller groups of closely related constructions, the Russian Constructicon project reaches a new level by attempting a more comprehensive classification. Classification reveals the intricate structure that binds constructions together in the grammar of a language.

The analysis of large groups of constructions makes it possible to discover overall patterns. Relationships among constructions are observed both hierarchically within the Assessment and Attitude networks as realized by families and clusters, as well as horizontally across all three levels of organization. Families are related to other families motivating clusters, clusters are related to other clusters motivating networks, and networks are also related to each other. Relationships are formed through transitional constructions with multiple allegiances, as well as through near-synonymy of constructions and families.

Within families there is some tendency for syntactic similarities as well. Overall we find a propensity for clausal constructions and constructions with the anchor in the role of adverbial modifier. When semantic and syntactic patterns are recognized, they can serve as the basis for further expansion of the constructicon. In other words, once we know what to look for, it becomes easier to identify additional candidates for inclusion in the constructicon. Thus the process of classification has directly facilitated the process of collection.

The distribution of data can serve to test and flesh out hypotheses made in previous scholarship regarding constructions and semantics. For example, construction grammarians (Goldberg, 1995, 2005; Croft, 2001; Langacker, 2008) have hypothesized that the grammar of an entire language consists of an interconnected system of constructions, hence the term “constructicon.” Our study gives detailed concrete evidence of the internal structure of a constructicon. Our study likewise lends support to the hypothesis formulated in previous scholarship (e.g., Arutjunova, 1988) regarding a greater number and diversity of linguistic means employed for encoding negative evaluation, which is what we find in our data.

## Data Availability Statement

The datasets presented in this study can be found in online repositories. The names of the repository/repositories and accession number(s) can be found below: https://site.uit.no/russian-constructicon.

## Author Contributions

Both authors listed have made a substantial, direct and intellectual contribution to the work, and approved it for publication.

## Conflict of Interest

The authors declare that the research was conducted in the absence of any commercial or financial relationships that could be construed as a potential conflict of interest. The reviewer SS declared a past collaboration with the author LJ to the handling editor.

## Footnotes

1 For details about abbreviations and our system of naming constructions see the Appendix.

2 It should be noted, however, that this construction has a specific prosodic contour that combines overall exclamatory intonation with additional stress on the verb. However, it is not clear whether this suprasegmental characteristic is necessarily associated with expressing dissatisfaction (and indignation in this case) rather than emphasizing the verb and the construction as a whole. We leave this issue for future investigation.

3 Although morphemes are certainly “conventional, learned form-meaning pairings” (Goldberg, 2013, p. 17) and therefore can be treated as constructions, for some scholars it is debatable whether morphemes represent constructions on their own. The influential approach of construction morphology proposed by Booij (2010) treats morphemes not as constructions in their own right but rather as constituents of morphological constructions. For example, the English derivational suffix -*able* is analyzed as part of the construction [V_tr_ -able] (where V_tr_ stands for a transitive verb that attaches the suffix -*able* to produce a deverbal adjective).

4 In such cases, the name of the construction indicates the anchor verb in the infinitive form and restricts its variation to the past tense: *najti-Pst.* For more details on our system of naming constructions see the Appendix.

5 This is the reason why the name of this construction does not indicate the standard subject position NP-Nom, as opposed to verb argument constructions with specific anchor verbs like *NP-Nom predstavljat’ iz sebja NP-Acc* (illustrated in Table 1).

6 Coercion effects can be observed in morphological constructions. Booij (2016, p. 429) argues that in the English [*un*-V]_V_ construction, the attachment of the prefix *un*- to stative verbs like *see* and *have* coerces these verbs to denote telic achievements, as observed in these examples from Bauer et al., 2013, p. 375, *And once you’ve seen it, you can never unsee it; The other big difference is once you have AIDS, you can’t unhave it.* Booij (2016, p. 429) points out that “it is the construction as a whole that imposes this interpretation of telic achievements on these *un-*verbs,” and this comports with his approach to morphemes as constituent parts of constructions.

7 See the Appendix for the list of abbreviations and explanation of how the names of constructions represent their morphosyntactic structure. Each slot and morphological specifications in the names of constructions are verified by data from the Russian National Corpus, supplemented by internet searches where data is sparse.

8 A detailed comparison of our radial category model of Assessment constructions with types proposed by von Wright (1963) and Arutjunova (1988) goes beyond the scope of this article.

9 We calculate this by adding the number of constructions from transitional families (3 + 5 + 9 + 12 + 18 = 47) and the number of constructions with multiple motivations inside the four clusters (26 + 53 + 88 + 28 + 6 + 47-224 = 24).

10 It seems unnatural to split the three *Importance* families of constructions depending on the animacy of the object they take. We can attribute thirteen constructions to *Importance/Worth* and fifteen constructions to *Importance/Power*, including nine constructions that can encode both.

11 These are classified as “interrogative/relative pronouns” (Wade, 1992, p. 126–133), the corresponding Russian term is “voprositel’no-otnositel’nye mestoimenija” (Padučeva, 2015, compare also “voprositel’nye/otnositel’nye mestoimenija” in the Russian National Corpus).

12 In this case, we treat *budeš’* not as a form of the auxiliary verb *byt’*, which is part of the analytic future tense form *budeš’ delat’ “*will do,” but as an optional “frozen” element of this construction.

13 We suggest that *nekuda devat’sja* is a periphrastic element that can only be used in the present tense. Adding a copula verb in past or future tense shifts the semantics of this expression to its literal meaning.

14 This construction can refer to emotional states even without naming them, as supported by corpus examples like *Emu bez morja i žizn’ ne v žizn’* “For him there is no joy in life without the sea [lit. life not in life]).”

## Appendix

In this study, we follow the representation of constructions in the Russian Constructicon. For each construction, we provide its name and a short illustration: e.g., *najti-Pst NP-Acc! Našli razvlečenie!* “What a bad way to amuse yourself! [lit. found amusement].”

The name of a construction is a short morphosyntactic formula that includes fixed lexical parts as well as grammatical slots indicated by means of common abbreviations: NP – noun phrase; VP – verb phrase; PP – prepositional phrase; XP – any phrasal unit (a slot that can be NP or VP or AP or PP); Adj – adjective; Adv – adverb; PronPers – personal pronoun; PronInt – interrogative/relative pronoun; PronPoss – possessive pronoun; Cl – clause; Short – short form. When necessary, we specify morphological characteristics of the fixed lexeme or a slot, where we use abbreviations according the Leipzig Glossing rules: Nom – Nominative case; Gen – Genitive case; Dat – Dative case; Acc – Accusative case; Loc – Locative case; Ins – Instrumental case; Sg – Singular; Pl – Plural; Pst – Past tense; Inf – Infinitive; Imp – Imperative; Ipfv – Imperfective verb; Pfv – Perfective verb; Cop – Copula; Pred – Predicative; ∼ – Reduplication. We combine these abbreviation systems, as in e.g., NP-Nom – Noun Phrase in the Nominative case. In our system of annotation, the symbol () indicates optional elements of a fixed part, and the symbol “/” is used to list alternative elements of construction. Each slot and morphological specifications are verified by the data from the Russian National Corpus, supplemented by internet searches where data is sparse.

In representing the syntactic structure of constructions, we adopt the following strategies. If a construction contains an NP that can be used not only in the predicative function marked with the nominative case but also in other roles (e.g., object, etc.) encoded with oblique cases, we do not specify the case in the construction name: e.g., *NP na nule* [lit. NP on zero], as in *Immunitet na nule* “Immunity is at the zero level” vs. *Vypisali bol*’*nogo s immunitetom na nule* “They released a patient with immunity at the zero level.”

If a construction contains an NP that is only used in the predicative function, we indicate its form as the default NP-Nom, as it appears with the present tense copula: e.g., *NP-Nom Cop NP-Nom VP-Inf* (as in *On master gotovit’* “He is good at cooking [lit. expert cook]”). We assume that the instrumental case marking of the predicative NP that appears with the past and/or future tense copula is a general rule of Russian grammar and this is mentioned in the commentary field on the Russian Constructicon website: e.g., *On byl masterom gotovit’* “He was good at cooking [lit. expert cook].” Note that we include the copula in the name of a construction only if the copula verb can be used in this construction not only in the present tense but also in other tense(s), as in this example.

Some constructions contain reduplicated nouns rather than NPs, and we represent this accordingly: e.g., *NP-Nom Cop vsem Noun-Dat.Pl* ∼*Noun-Nom* (as in *Vsem borščam boršč* “The best vegetable soup of all [lit. to all soups soup]).”

In verb argument constructions that contain a specific verb lexeme (the anchor verb) and slots for the verb’s arguments, we specify the subject slot even if it has a default nominative case marking: e.g., *NP-Nom znat’ tolk v NP-Loc* (as in *On znaet tolk v nastol’nyx igrax “*He is an expert in board-games [lit. He knows sense in board-games]”). Normally, the anchor verb is given in the infinitive to represent any inflectional form. For example, in the construction *NP-Nom znat’ tolk v NP-Loc*, the infinitive of the anchor verb *znat*’ “know” indicates that this verb can be used in this construction in other forms too.

If the anchor verb can be used in a construction only in a specific grammatical form, the construction name indicates this specific form (or forms, if there are very few options): e.g., *s PronPers-Gen xvatit/xvatilo (NP-Gen)*, as in *S menja xvatit* “I’m fed up [lit. from me enough].” If the use of the anchor verb in the construction is restricted to a certain sub-paradigm, this is indicated accordingly. For example, in the construction *najti-Pst NP-Acc!* (as in *Našli razvlečenie!* “What a bad way to amuse yourself! [lit. found amusement]”), the anchor verb *najti* ‘find’ can appear only in the past tense.

For constructions that contain a VP, we do not include the subject slot NP-Nom in the name of the construction, because the case marking of the arguments (including the logical subject) depends on specific verb lexemes: compare *večno VP* in *Večno mne ne vezet* “I am always short on luck [lit. eternally to me not catch-luck]” (where the logical subject has an experiencer role and is marked with the dative case) vs. *Večno Petr opazdyvaet “*Peter is always late [lit. eternally Peter is late]” (where the logical subject has the agent role and is encoded with the nominative case).
